# Ischemic Postconditioning Protects Aged Myocardium Against Ischemia/Reperfusion Injury by Regulating Mitochondrial Dynamics via piR‐4913

**DOI:** 10.1002/advs.78464

**Published:** 2026-10-28

**Authors:** Xiangyu Zhao, Yanchun Li, Nan Li, Juan Zhang, Xiaolong Ma, Fei Yang, Ruihan Long, Yining Tian, Yichen Wang, Shengchao Ma, Huiping Zhang, Yideng Jiang

**Affiliations:** ^1^ NHC Key Laboratory of Metabolic Cardiovascular Diseases Research Ningxia Medical University Yinchuan China; ^2^ School of Basic Medical Sciences Ningxia Medical University Yinchuan China; ^3^ Ningxia Key Laboratory of Vascular Injury and Repair Research Ningxia Medical University Yinchuan China; ^4^ Department of Medical Genetics Hunan Provincial Maternal and Child Health Care Hospital Changsha China; ^5^ Medical Experimental Center General Hospital of Ningxia Medical University Yinchuan China

**Keywords:** Drp1, ischemic postconditioning, O‐GlcNAcylation, piRNA

## Abstract

Ischemic postconditioning (IPostC) mitigates myocardial ischemia/reperfusion (I/R) injury, but its regulation of mitochondrial dynamics in the aged heart remains unclear. This study investigates the underlying mechanisms by evaluating PIWI‐interacting RNA (piRNA) expression profiles after I/R injury. Functional assays using piR‐4913 silencing assess its impact on infarct size, mitochondrial ATP production, and oxidative stress. Mechanistic analyses identify that piR‐4913, a cardiac‐enriched piRNA markedly upregulated in aged hearts following I/R injury, is effectively suppressed by IPostC. Silencing piR‐4913 confers cardioprotection by limiting infarct size, enhancing ATP production, and reducing oxidative stress. Mechanistically, piR‐4913 acts as a key facilitator that promotes the recruitment of OGT to Drp1, thereby catalyzing the O‐GlcNAcylation modification of Drp1 at Thr604. This modification promotes Drp1 mitochondrial translocation and Fis1 interaction, driving excessive mitochondrial fission and dysfunction. In vivo validation using a Drp1‐T604A mutant demonstrates profound resistance to I/R injury, confirming the critical role of Thr604 O‐GlcNAcylation in age‐related cardiac damage. Collectively, IPostC exerts cardioprotection in the aging heart by restraining the piR‐4913/OGT/Drp1 axis, thereby restoring mitochondrial proteostasis and dynamics. This pathway represents a promising therapeutic target to improve clinical outcomes in elderly patients with myocardial infarction.

## Graphical abstract

A tiny RNA tips the scales of cardiac injury in aging. piR‐4913 recruits OGT to Drp1 and drives its O‐GlcNAcylation at Thr604, promoting excessive mitochondrial fission that aggravates ischemia‐reperfusion injury. Ischemic postconditioning silences this pathway to restore mitochondrial balance and protect the aged myocardium, revealing piR‐4913 as a promising therapeutic target.

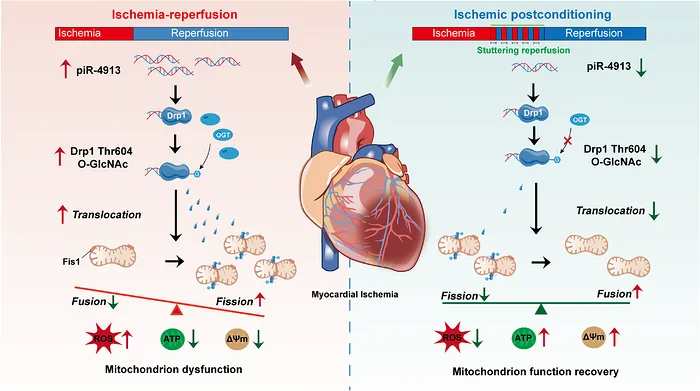

**Abbreviations**
±dP/dt_max_
: The maximum rates of pressure rise and decline
AAV9: Adeno‐associated virus 9
CHX: Cycloheximide
Drp1: Dynamin‐related protein 1
EF: Ejection fraction
FS: Fractional shortening
H/R: Hypoxia/reoxygenation
HPostC: Hypoxic postconditioning
I/R: Ischemia/reperfusion
IPostC: Ischemic postconditioning
LVDP: Left ventricular diastolic pressure
LVSP: Left ventricular systolic pressure
MS: Mass spectrometry
OGT: O‐GlcNAc transferase
piRNAs: PIWI‐interacting RNAs
PTM: Post‐translational modification
RIP: RNA immunoprecipitation
RNA‐seq: RNA sequencing
TEM: Transmission electron microscopy

## 1 Introduction

Ischemic postconditioning (IPostC), characterized by brief, intermittent cycles of ischemia and reperfusion applied at the onset of revascularization, remains a pivotal strategy for mitigating myocardial ischemia/reperfusion (I/R) injury [1, 2]. In the elderly, degenerative structural alterations and metabolic dysfunction significantly increase myocardial susceptibility to I/R challenge, with over 80% of infarction‐related deaths occurring in individuals aged ≥ 65 years [3, 4]. Despite its clinical importance, the role of IPostC in regulating mitochondrial dynamics, a cornerstone of myocardial homeostasis [5], in the aged heart remains largely elusive.

Mitochondria, the “powerhouses” of the cell, maintain their morphology and functional integrity through a finely tuned balance between fusion and fission [6, 7]. Evidence suggests that excessive mitochondrial fission is the Achilles’ heel of the aged myocardium during I/R [8, 9]. Thus, elucidating how IPostC preserves this dynamic equilibrium in the elderly is essential for developing novel therapeutic interventions.

PIWI‐interacting RNAs (piRNAs), a class of small non‐coding RNAs (24–31 nt), were traditionally viewed as guardians of the germline through transposon silencing [10]. However, emerging evidence has revolutionized our understanding of piRNAs, revealing their diverse roles in somatic tissues, particularly in stress‐responsive diseases such as cancer and cardiovascular disorders [11, 12]. Recent studies highlight the potential of piRNAs as critical regulators of gene expression via epigenetic modulation and post‐translational control. For instance, piR‐HAAPIR has been shown to exacerbate myocardial apoptosis by stabilizing mRNA through N4‐acetylcytidine modification [13]. Given that I/R injury is fundamentally a dynamic process of hypoxia and reoxygenation, we hypothesized that specific piRNAs may form a regulatory network that dictates the resilience of the aged heart to I/R stress.

O‐GlcNAcylation, a reversible post‐translational modification (PTM) catalyzed by O‐GlcNAc transferase (OGT), serves as a sophisticated cellular nutrient and stress sensor [14, 15]. Unlike other PTMs, O‐GlcNAcylation rapidly fluctuates to maintain protein homeostasis under pathological conditions, such as oxidative stress and hypoxia. In the cardiovascular system, while acute O‐GlcNAc elevation may be cardioprotective, chronic or aberrant modification often worsens injury by impairing contractile apparatus function [16]. Notably, Dynamin‐related protein 1 (Drp1), the master regulator of mitochondrial fission, is highly susceptible to O‐GlcNAcylation. In models of chronic hypoxia, Drp1 O‐GlcNAcylation has been shown to synergize with phosphorylation to stabilize its mitochondrial localization, thereby driving pathological remodeling [17]. Yet, whether IPostC maneuvers Drp1 O‐GlcNAcylation to protect the senescent myocardium remains an enigma.

In the present study, we identify a novel mechanism by which IPostC confers cardioprotection in the aged heart by restraining excessive mitochondrial fission. We characterized a novel cardiac‐enriched piRNA, piR‐4913, which is pathologically upregulated during I/R but normalized by IPostC. Mechanistically, piR‐4913 binds to Drp1 and recruits OGT to catalyze O‐GlcNAcylation of Drp1 at Threonine 604 (Thr604). This modification functions as a “molecular switch” that triggers Drp1 mitochondrial translocation and excessive fission. Our findings provide the first evidence that targeting the piR‐4913/OGT/Drp1 axis, specifically at the Thr604 residue, represents a novel therapeutic strategy for preserving myocardial function in the aging population.

## 2 Materials and Methods

### 2.1 Animal Model Establishment

Aged male Sprague‐Dawley rats (22–24 months old, weight 600 ± 100 g) were used in this study. The animals were obtained from the Experimental Animal Center of Ningxia Medical University and maintained in a specific pathogen‐free (SPF) facility (22 ± 2°C). Following an 8‐h fast, rats were anesthetized via intraperitoneal injection of 2% pentobarbital sodium (30 mg/kg), with anesthesia depth confirmed by the loss of the toe‐pinch reflex.

Under continuous electrocardiographic (ECG) monitoring (standard lead II), tracheal intubation was performed, and the rats were connected to a small animal ventilator to maintain respiratory function. Core body temperature was strictly maintained at 37 ± 0.5°C using a heating pad. A left thoracotomy was performed at the third‐fourth intercostal space to expose the heart. The left anterior descending coronary artery (LAD) was ligated using a 5‐0 silk suture passed beneath the left atrial appendage and the pulmonary conus. Myocardial ischemia was confirmed by ST‐segment elevation or prominent T‐wave elevation on the ECG.

The ischemia/reperfusion (I/R) model was established by 30 min of LAD occlusion followed by 180 min of reperfusion. For the ischemic postconditioning (IPostC) group, three cycles of 10‐s reperfusion and 10‐s re‐occlusion were applied immediately after the 30‐min ischemic period, followed by full reperfusion. All experimental protocols were approved by the Animal Care and Use Committee of Ningxia Medical University (Ethics No. 2023‐G109) and complied with the institutional guidelines for animal welfare.

### 2.2 Cell Culture and Models

#### 2.2.1 Cell Culture and Senescence Induction

Rat cardiomyocyte‐derived H9c2 cells (Procell, China) were cultured in DMEM (Gibco, USA) supplemented with 10% FBS (Invitrogen, USA) and 100 U/mL penicillin–streptomycin (Gibco, USA). To establish the aged cardiomyocyte model, H9c2 cells were treated with 8 mg/mL D‐galactose (MCE, USA) in complete culture medium for 9 consecutive days.

#### 2.2.2 H/R and HPostC Modeling

For the hypoxia/reoxygenation (H/R) model, aged cardiomyocytes were placed in an anaerobic chamber for 180 min of hypoxia, followed by 120 min of reoxygenation under normoxic conditions. For hypoxia postconditioning (HPostC), aged cardiomyocytes were subjected to three cycles of 5‐min reoxygenation and 5‐min hypoxia immediately after the initial 180‐min hypoxia period, followed by 120 min of full reoxygenation.

### 2.3 Statistical Analysis

Quantitative analyses were performed using values derived from independent biological replicates, and the data are presented as the mean ± Standard Error of the Mean (SEM). Statistical analyses were performed using GraphPad Prism 8.0 software (GraphPad Software, San Diego, CA, USA). Comparisons between two groups were evaluated by unpaired Student's t‐test; multiple‐group comparisons were analyzed by one‐way ANOVA followed by Tukey's post‐hoc test. A p‐value < 0.05 was considered to indicate a statistically significant difference.

## 3 Results

### 3.1 IPostC Restores Mitochondrial Dynamic Balance to Prevent I/R Injury of Aged Myocardium

IPostC is an effective strategy to reduce myocardial I/R injury. To investigate its protective effects on aged myocardium, we established integrated in vivo and in vitro models. In vitro, cardiomyocytes were treated with 8 mg/ml D‐galactose for 9 days to establish in vitro models of aged cardiomyocytes under Normoxia, H/R, and HPostC conditions. In vivo, aged rats were subjected to Sham, I/R, and IPostC procedures to establish the animal model [1]. Electrocardiograms (ECGs) revealed that the ST segment in lead II was elevated during the ischemic phase and returned to baseline levels within 15 min of reperfusion, confirming the successful establishment of aged myocardial I/R injury (Figure S1A,B). Hemodynamic assessment revealed that IPostC significantly attenuated I/R‐induced impairments in left ventricular systolic pressure (LVSP), left ventricular diastolic pressure (LVDP), and the maximum rates of pressure rise and decline (± dP/dt_max_) (Figure 1A). These functional improvements were further validated by echocardiography, which showed a marked preservation of left ventricular ejection fraction (EF) and fractional shortening (FS) in the IPostC‐treated group compared to the I/R group (Figure 1B,C). Consistent with the functional data, IPostC treatment resulted in significantly lower serum levels of myocardial injury biomarkers LDH, CK‐MB, and cTn‐I, confirming a reduction in cardiomyocyte damage in aged hearts (Figure 1D). Subcellular analysis via transmission electron microscopy (TEM) demonstrated that I/R injury triggered extensive mitochondrial fragmentation in the aged myocardium, evidenced by a reduced average mitochondrial area and an increased fission frequency (Figure 1E,F). Notably, IPostC effectively preserved mitochondrial morphology by rebalancing mitochondrial dynamics. This was supported by Western blot analysis, where IPostC reversed the I/R‐induced dysregulation of key fission (Drp1, Fis1) and fusion (Mfn1, Mfn2, and Opa1) proteins (Figure 1G). Furthermore, JC‐1 fluorescence staining was employed to assess mitochondrial membrane potential (MMP) in the aged myocardium. The red/green fluorescence intensity ratio, a well‐established marker of MMP, was significantly reduced following I/R injury but effectively restored by IPostC (Figure S1C). Parallel in vitro experiments showed that hypoxic postconditioning (HPostC) markedly reduced the increase of LDH, CK‐MB, and cTn‐I levels in the culture supernatants of aged cardiomyocytes under H/R conditions, accompanied by a notable increase in cell viability (Figure S1D,E). Additionally, H/R decreased mitochondrial area and compromised mitochondrial membrane potential (MMP) integrity (Figure S1F), while simultaneously promoting mitochondrial fission (Figure 1H); however, these effects were significantly attenuated by HPostC. Collectively, these findings demonstrate that IPostC confers robust cardioprotection in the aged heart, primarily by inhibiting excessive mitochondrial fission.

**FIGURE 1:**
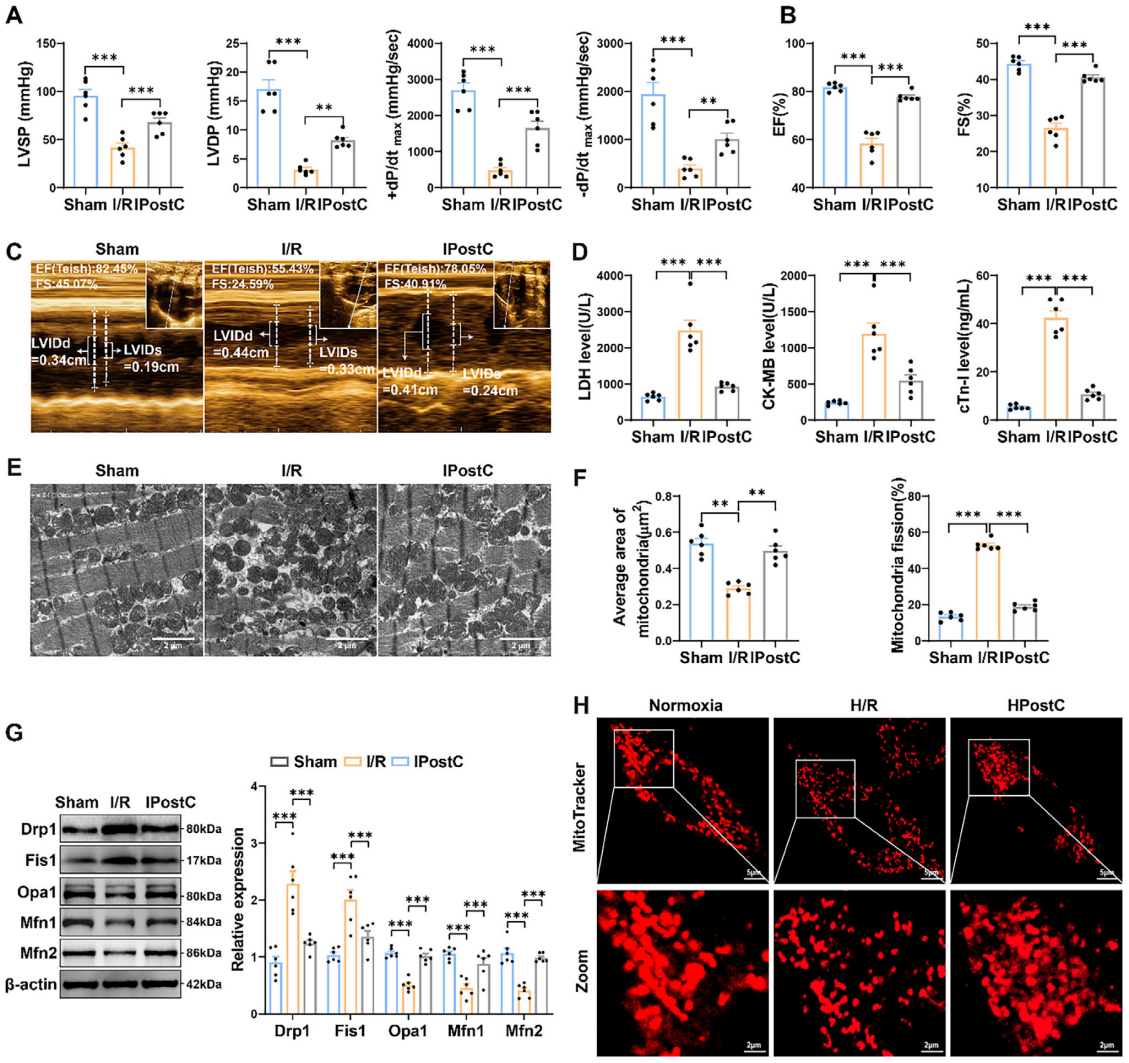
IPostC attenuates I/R injury by inhibiting excessive mitochondrial fission in aged hearts. Aged rats were subjected to Sham, I/R, and IPostC protocols (*n* = 6). (A) Assessment of cardiac hemodynamics, including left ventricular systolic pressure (LVSP), left ventricular diastolic pressure (LVDP), and the maximum rate of pressure rise/fall (±dP/dt_max_) recorded via the BL‐420N system. (B, C) Representative M‐mode echocardiograms and quantitative analysis of left ventricular ejection fraction (EF) and fractional shortening (FS). (D) Serum levels of lactate dehydrogenase (LDH), creatine kinase‐MB (CK‐MB), and cardiac troponin I (cTn‐I) measured by an automated biochemical analyzer. (E, F) Representative transmission electron microscopy (TEM) images showing mitochondrial ultrastructure in the myocardium. Scale bar = 2 µm. (G) Western blot analysis of mitochondrial fission‐related proteins (Drp1, Fis1) and fusion‐related proteins (Mfn1, Mfn2, OPA1) in aged myocardium. (H) Representative images of mitochondrial morphology labeled with MitoTracker Red. Scale bar = 5 µm. Data are mean±SEM. ^**^ *p* < 0.01, ^***^ *p* < 0.001.

### 3.2 Identification of piRNAs in IPostC That Mitigate I/R Injury in Aged Myocardium

piRNA, as an emerging type of small non‐coding RNA (ncRNA), possesses characteristics such as high stability, conservation, and tissue specificity. Currently, research on the role of piRNA in human diseases, apart from reproductive system diseases, mainly focuses on cancers, heart diseases, and neurological disorders. Oxygen sensitivity is a common feature of the tissues in these three diseases, and ischemia/reperfusion essentially represents an alteration of hypoxia‐reoxygenation. Therefore, we hypothesize that piRNA is also involved in the I/R injury of aged myocardium. First, we performed RNA sequencing (RNA‐seq) on aged cardiomyocytes subjected to Normoxia, H/R, and HPostC. The sequenced piRNAs ranged from 21 to 32 nt in length, with a characteristic peak at 26 nt (Figure 2A). These piRNAs were predominantly derived from intergenic repetitive elements and were distributed across all chromosomes, with the highest density on chromosome 4 (Figure S2A,B). Notably, the identified piRNAs exhibited a strong 5’‐uracil (5’U), particularly within the 25–32 nt fraction, consistent with canonical piRNA features (Figure 2B and Figure S2C). We next performed hierarchical cluster analysis on the expression levels of the differentially expressed piRNAs (Figure 2C). Differential expression analysis was performed using DESeq2, with |FC| > 2 and *p* < 0.05 as the screening criteria. Compared with the Normoxia group, there were 39 differentially expressed piRNAs in the H/R group, including 13 up‐regulated piRNAs and 26 down‐regulated piRNAs. A total of 63 differentially expressed piRNAs were identified in the HPostC group compared with the H/R group, of which 48 piRNAs were up‐regulated and 15 piRNAs were down‐regulated (Figure 2D). Venn diagram and heatmap analyses further narrowed down the candidate piRNAs associated with HPostC‐mediated recovery (Figure 2E,F). GO and KEGG enrichment analyses indicated that these overlapping piRNAs are primarily involved in membrane‐bound organelle function, protein binding/localization, and metabolic pathways, including the AMPK pathway, suggesting a potential role in mitochondrial regulation (Figure 2G,H).

**FIGURE 2:**
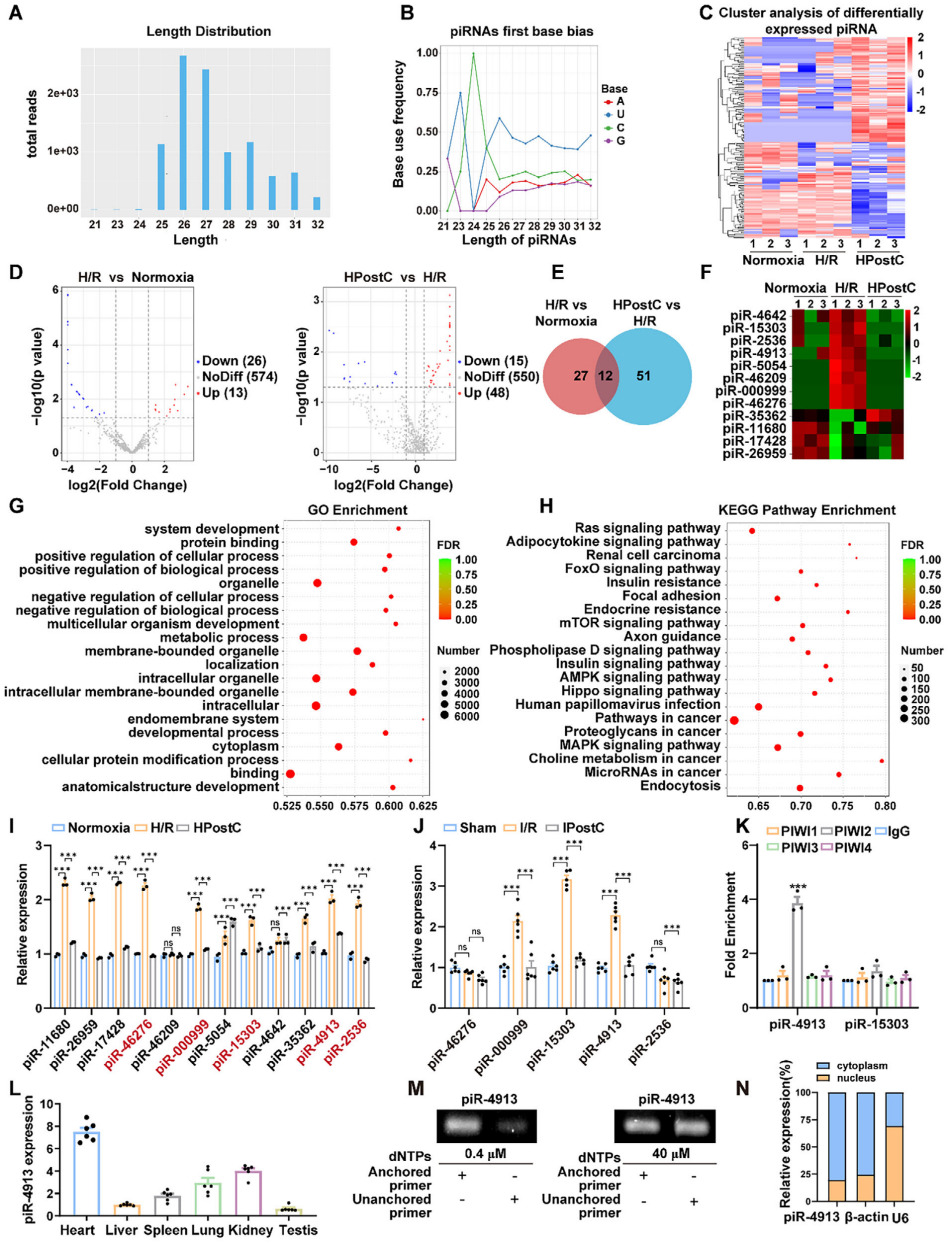
Identification and characterization of piR‐4913 in IPostC‐mediated protection against I/R injury in aged hearts. (A) Sequence length distribution of all detected piRNAs in aged cardiomyocytes. (B) First‐nucleotide bias of piRNAs across different length cohorts. (C) Hierarchical clustering heatmap of the union of differentially expressed piRNA clusters identified in different groups. Expression values are presented as row‐wise Z‐scores calculated from log10(TPM+1)‐transformed TPM values. Red and blue indicate up‐ and down‐regulated piRNAs, respectively (*n* = 3). (D) Volcano plots showing DE‐piRNAs in aged cardiomyocytes under H/R and HPostC conditions. (E) Venn diagram illustrating the intersection of DE‐piRNAs across treatment groups. (F) Heatmap displaying the expression profiles of 12 candidate DE‐piRNAs in the aged myocardium. Red: upregulated; Green: downregulated. (G, H) GO and KEGG pathway enrichment analyses of the 12 candidate DE‐piRNAs. (I, J) qRT‐PCR validation of the 12 candidate piRNAs in aged cardiomyocytes (HPostC) and aged myocardium (IPostC) (*n* = 3–6). (K) Interaction between candidate piRNAs and PIWI proteins assessed by RNA immunoprecipitation (RIP) assay (*n* = 3). (L) Tissue‐specific expression profile of piR‐4913 in aged rats determined by qRT‐PCR (*n* = 6). (M) Verification of the 3'‐end 2'‐O‐methylation of piR‐4913 using the RTL‐P (Reverse Transcription at Low dNTP concentrations followed by PCR) assay. RT‐PCR results using unanchored or anchored primers at varying dNTP concentrations. (N) Subcellular localization of piR‐4913 in aged cardiomyocytes analyzed by qRT‐PCR in nuclear and cytoplasmic fractions. GAPDH and U6 were used as cytoplasmic and nuclear markers, respectively. Data are presented as mean±SEM. ^**^ *p* < 0.01, ^***^ *p* < 0.001.

We next validated the expression of candidate piRNAs using qRT‐PCR. Five piRNAs showed expression patterns consistent with the RNA‐seq data in aged cardiomyocytes (Figure 2I). However, only piR‐000999, piR‐15303, and piR‐4913 were significantly altered in the aged myocardium in vivo (Figure 2J). Given that piR‐000999 has been previously characterized as testis‐specific [18], we focused on piR‐15303 and piR‐4913. RNA immunoprecipitation (RIP) assays demonstrated that only piR‐4913 specifically interacted with the PIWIL2 protein (Figure 2K and Figure S3). Tissue distribution analysis revealed that piR‐4913 is highly enriched in the heart (Figure 2L). To further characterize piR‐4913, we assessed its 3′‐terminal 2′‐O‐methylation status using the RTL‐P assay, which exploits the inhibitory effect of 2′‐O‐methylation on reverse transcription under limiting dNTP conditions. In this assay, 3′‐terminal 2′‐O‐methylation is expected to result in reduced RT‐PCR amplification with the unanchored primer relative to the anchored primer at a low dNTP concentration (0.4 µM), whereas this difference is attenuated at a higher dNTP concentration (40 µM). The RTL‐P results were consistent with this characteristic response, supporting the presence of 3′‐terminal 2′‐O‐methylation, a hallmark of mature piRNAs (Figure 2M). piR‐4913 was also evolutionarily conserved across multiple species, including rats, mice, rabbits, pigs, pandas, and humans (Figure S2D). Subcellular fractionation and RNA‐FISH revealed that piR‐4913 was localized predominantly in the cytoplasm of aged cardiomyocytes (Figure 2N and Figure S2E). Collectively, these findings identify piR‐4913 as a novel, cardiac‐enriched, PIWI‐bound piRNA that may regulate mitochondrial function during IPostC‐mediated protection against I/R injury in aged myocardium.

### 3.3 Inhibition of piR‐4913 Alleviates I/R Injury in Aged Myocardium by Suppressing Mitochondrial Fission

To elucidate the functional role of piR‐4913 in mitochondrial dynamics during HPostC, aged cardiomyocytes were transfected with a piR‐4913 antagomir under Normoxia or H/R conditions (Figure S4A,B). Western blot analysis demonstrated that piR‐4913 knockdown significantly downregulated the pro‐fission proteins Drp1 and Fis1, while concurrently elevating the levels of fusion‐related markers OPA1, Mfn1, and Mfn2 (Figure 3A). In alignment with these molecular changes, confocal microscopy revealed that piR‐4913 inhibition restored mitochondrial morphology, characterized by an increased average mitochondrial area and a reduced mitochondrial fission index (Figure 3B). Conversely, overexpression of piR‐4913 via an agomir exacerbated mitochondrial fragmentation, as evidenced by skewed fission/fusion protein expression and disrupted mitochondrial architecture (Figure S4C and Figure 3C,D). In addition, under normoxic conditions, piR‐4913 knockdown had no significant effect on mitochondrial fission (Figure S5A,B).

**FIGURE 3:**
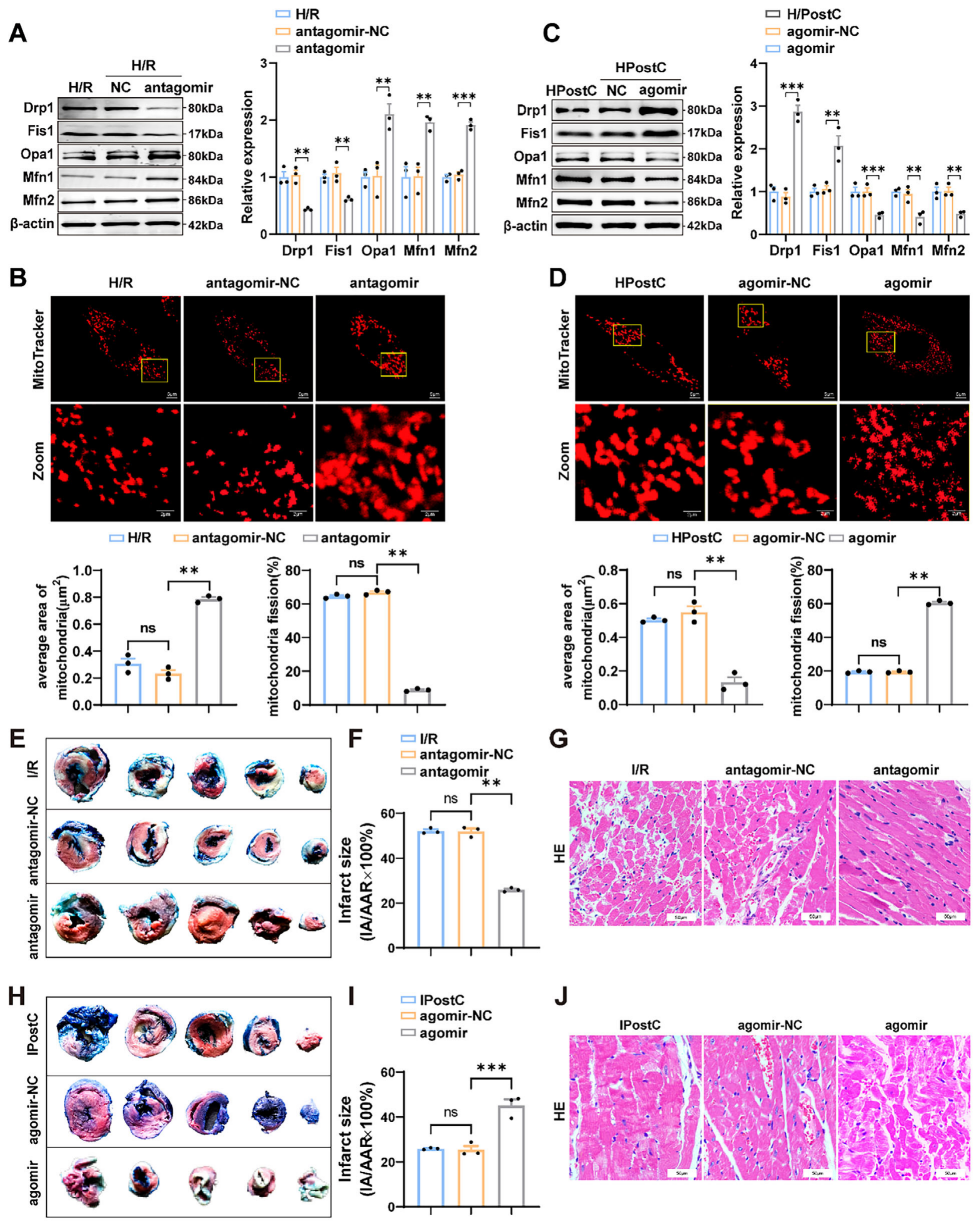
piR‐4913 inhibition mediates the cardioprotective effects of IPostC by regulating mitochondrial dynamics. (A) Western blot analysis of mitochondrial fission proteins (Drp1, Fis1) and fusion proteins (OPA1, MFN1, MFN2) in aged cardiomyocytes transfected with piR‐4913 antagomir under H/R conditions (*n* = 3). (B) Representative images and quantification of mitochondrial morphology using MitoTracker Red (*n* = 3). Scale bars = 5 µm. (C, D) Western blot analysis of fission/fusion proteins and representative MitoTracker Red staining in aged cardiomyocytes transfected with piR‐4913 agomir under HPostC conditions (*n* = 3). (E, F) Representative images and quantitative analysis of myocardial infarct size in aged rats following piR‐4913 antagomir treatment and I/R injury, determined by Evans blue and TTC double staining. (G) Histological changes in myocardial tissues visualized by HE staining (light microscopy). (H–J) Evans blue/TTC double staining and HE staining of myocardial infarction and pathological changes following piR‐4913 agomir treatment under IPostC conditions in aged rats. Data are expressed as mean±SEM. ^**^ *p* < 0.01, ^***^ *p* < 0.001.

To validate these findings in vivo, piR‐4913 antagomir or piR‐4913 agomir was administered to aged rats that had undergone I/R injury or IPostC treatment (Figure S4D,E). Evans blue/TTC staining confirmed a substantial reduction in myocardial infarct size following antagomir administration (Figure 3E,F and Figure S7A). Furthermore, histological assessment via HE staining showed preserved myocardial structure with diminished inflammatory infiltration compared to the control groups (Figure 3G). Moreover, portable color Doppler ultrasound revealed that intravenous administration of the piR‐4913 agomir significantly attenuated left ventricular ejection fraction and fractional shortening following IPostC (Figure S6A,B). Consistently, serum levels of LDH, CK‐MB, and cTn‐I were significantly increased (Figure S6C), indicating that piR‐4913 abrogated the cardioprotective effects of IPostC. Evans blue/TTC dual staining further demonstrated that piR‐4913 agomir administration significantly increased myocardial infarct size in aged rats subjected to IPostC (Figure 3H,I and Figure S7B). HE staining corroborated these findings, revealing exacerbated structural disorganization and myocyte damage in the aged myocardium under IPostC conditions (Figure 3J). Additionally, piR‐4913 agomir induced excessive mitochondrial fission in cardiomyocytes (Figure S6D–F). Collectively, these data suggest that targeting piR‐4913 represents a potent therapeutic strategy to protect the aged heart against I/R injury, potentially serving as a molecular alternative to IPostC.

### 3.4 piR‐4913 Interacts With Drp1 and Promotes its Mitochondrial Translocation During IPostC

Given that piRNAs regulate intracellular gene expression by interacting with RNA‐binding proteins,^16^ we employed biotinylated sense and antisense piR‐4913 probes to identify potential protein partners involved in mitochondrial fission. Cell lysates from aged cardiomyocytes were subjected to RNA pull‐down followed by mass spectrometry (MS) analysis. Applying stringent filtering criteria (FC≥ 10 and *P* ≤ 0.05), we identified 43 proteins significantly enriched by the sense piR‐4913 probe compared to the antisense control (Figure 4A). Notably, Drp1 emerged as a top candidate, exhibiting the highest enrichment and MS identification scores (Figure 4B).

**FIGURE 4:**
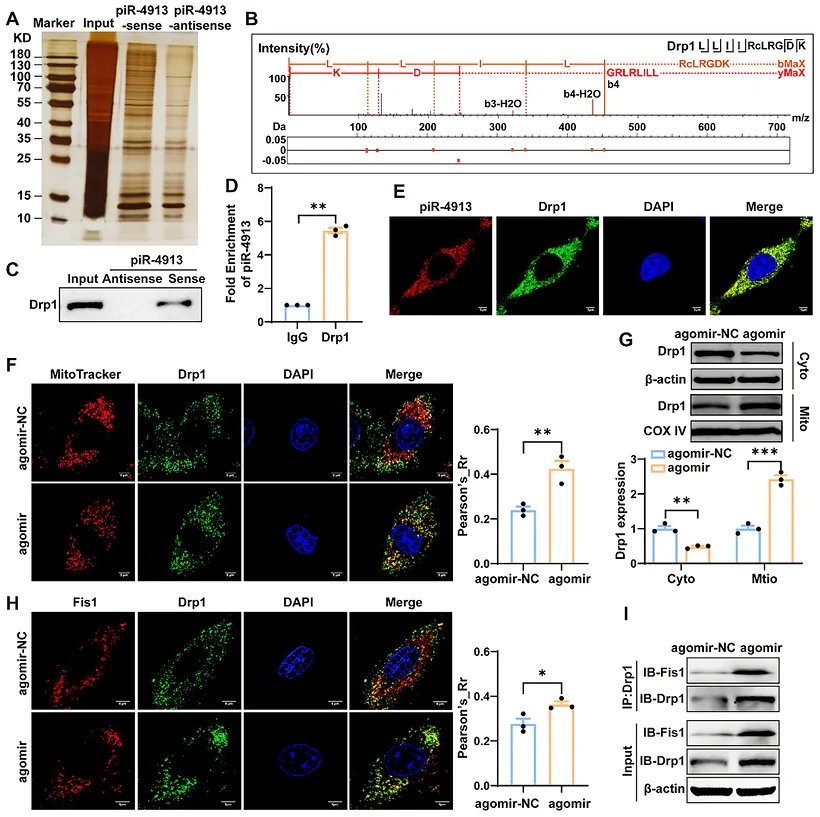
piR‐4913 interacts with Drp1 and promotes its mitochondrial translocation during HPostC. (A) Representative silver‐stained SDS‐PAGE gel showing proteins pulled down by biotinylated sense or antisense piR‐4913 probes in aged cardiomyocytes. Rectangular boxes indicate gel regions excised for mass spectrometry (MS) identification of the piR‐4913 interactome. (B) MS analysis showing the identification peptide peaks and enrichment scores of Drp1. (C) Validation of the interaction between piR‐4913 and Drp1 by Western blot analysis following RNA pull‐down. (D) RNA immunoprecipitation (RIP) assay using anti‐Drp1 or control IgG antibodies; piR‐4913 enrichment was quantified by qRT‐PCR (*n* = 3). (E) Representative RNA‐FISH images showing the subcellular co‐localization of piR‐4913 (red) and Drp1 (green) in aged cardiomyocytes. Nuclei were counterstained with DAPI. (F–I) Effects of piR‐4913 agomir transfection on Drp1 dynamics under HPostC conditions: (F) Representative confocal images showing co‐localization of Drp1 (green) with mitochondria (MitoTracker Red) (*n* = 6). (G) Subcellular fractionation and Western blot analysis of Drp1 expression in mitochondrial and cytosolic fractions (*n* = 3). (H) Immunofluorescence staining showing the co‐localization of Drp1 (green) and Fis1 (red) (*n* = 6). (I) Co‐immunoprecipitation (Co‐IP) analysis of the physical interaction between Drp1 and Fis1. Data are presented as mean±SEM. ^*^ *p* < 0.05, ^**^ *p* < 0.01, ^***^ *p* < 0.001.

To validate this interaction, RNA pull‐down assays confirmed the robust and specific binding of piR‐4913 to Drp1 (Figure 4C). This was further supported by RIP coupled with qRT‐PCR, which showed significant enrichment of piR‐4913 in Drp1‐containing complexes compared to the IgG control (Figure 4D). Additionally, RNA‐FISH combined with immunofluorescence revealed extensive co‐localization of piR‐4913 and Drp1, characterized by overlapping signals in the cytoplasm of aged cardiomyocytes (Figure 4E).

We next investigated whether piR‐4913 modulates the subcellular redistribution of Drp1 under HPostC conditions. Confocal microscopy demonstrated that piR‐4913 overexpression via agomir transfection significantly enhanced the recruitment of Drp1 to mitochondria, as evidenced by increased co‐localization with MitoTracker Deep Red (Figure 4F). Subcellular fractionation and western blot analysis corroborated these findings, showing decreased cytosolic Drp1 levels and a concomitant increase in mitochondrial Drp1 levels following piR‐4913 agomir treatment (Figure 4G). Furthermore, as Drp1 recruitment to mitochondria is typically facilitated by the docking protein Fis1, we assessed their interaction. Immunofluorescence assays showed heightened co‐localization between Drp1 and Fis1 upon piR‐4913 overexpression (Figure 4H). Co‐IP analysis further confirmed that piR‐4913 agomir treatment significantly strengthened the association between Fis1 and Drp1 (Figure 4I). Collectively, these data indicate that piR‐4913 promotes the mitochondrial translocation of Drp1 and its interaction with Fis1, thereby driving mitochondrial fission.

### 3.5 piR‐4913 Stabilizes Drp1 by Promoting O‐GlcNAcylation and Inhibiting Ubiquitin‐Mediated Degradation

To explore potential PTMs of Drp1, we conducted a bioinformatic analysis using the UniProt database, which indicated that Drp1 undergoes multiple PTMs, including phosphorylation, ubiquitination, SUMOylation, and O‐GlcNAcylation (Figure 5A). To test the regulatory role of piR‐4913, aged cardiomyocytes were transfected with a piR‐4913 agomir. Immunoprecipitation (IP) assays revealed that piR‐4913 overexpression significantly enhanced the O‐GlcNAcylation of Drp1 while concurrently suppressing its ubiquitination. Notably, no significant alterations were observed in Drp1 phosphorylation or SUMOylation (Figure 5B). Validation using exogenous Flag‐tagged Drp1 confirmed these findings, showing an O‐GlcNAcylation‐rich and ubiquitination‐poor profile consistent with the endogenous Drp1 results (Figure 5C).

**FIGURE 5:**
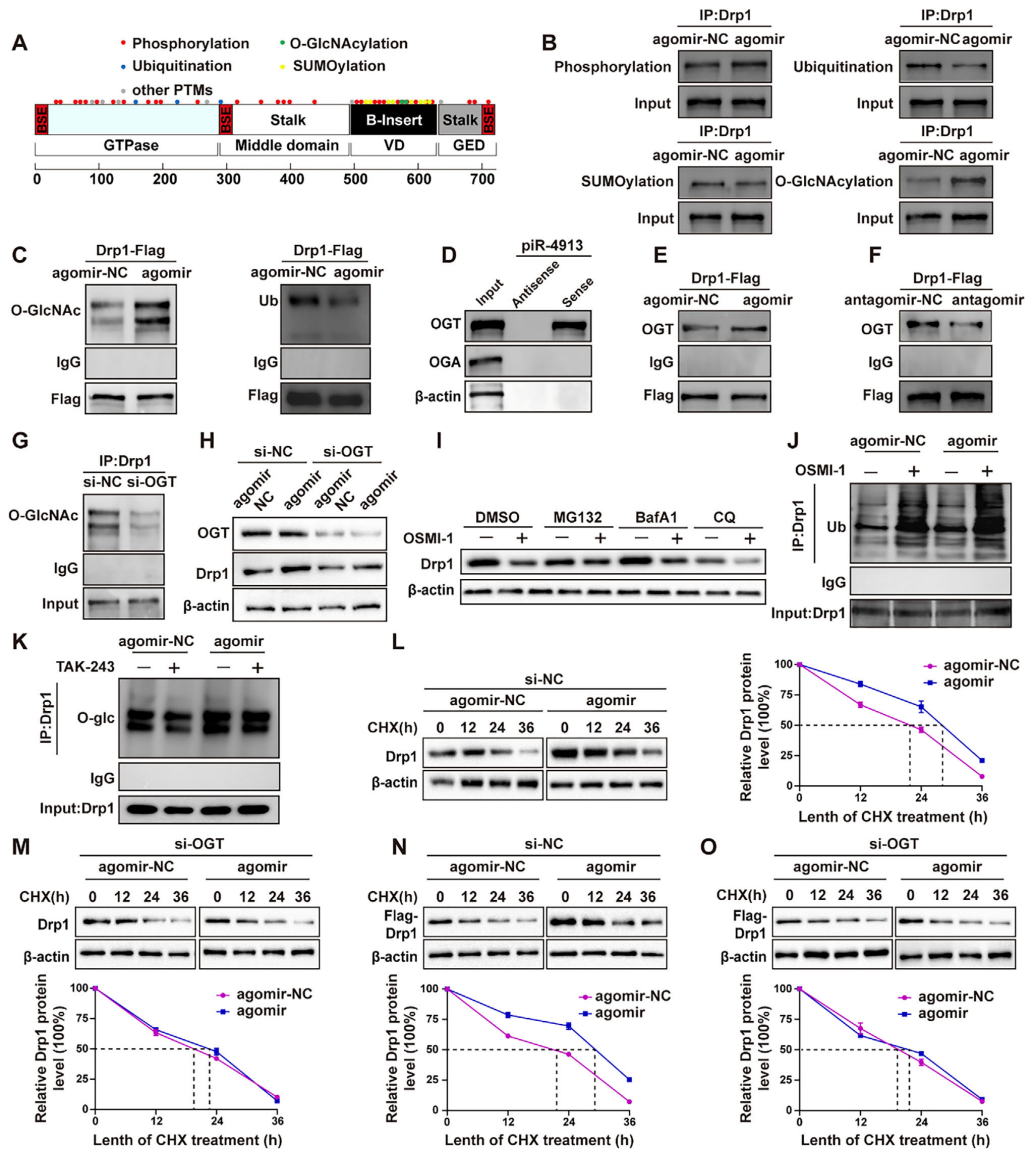
piR‐4913 stabilizes Drp1 by promoting O‐GlcNAcylation and inhibiting its ubiquitin‐mediated degradation. (A) Prediction of potential post‐translational modifications (PTMs) of Drp1 using the UniProt database. (B) Immunoprecipitation (IP) analysis of Drp1 phosphorylation, ubiquitination, SUMOylation, and O‐GlcNAcylation levels in aged cardiomyocytes transfected with piR‐4913 agomir under HPostC conditions. (C) IP and immunoblotting of O‐GlcNAcylation and ubiquitination levels in aged cardiomyocytes co‐transfected with Flag‐Drp1 and/or piR‐4913 agomir. (D) RNA pull‐down assay using biotinylated piR‐4913 or negative control (NC) probes, followed by Western blot analysis of OGT and OGA. (E, F) Co‐immunoprecipitation analysis of the physical interaction between Drp1 and OGT in aged cardiomyocytes transfected with piR‐4913 agomir or piR‐4913 antagomir under HPostC or H/R conditions. (G, H) Effect of OGT knockdown on Drp1: (G) Drp1 O‐GlcNAcylation levels in si‐OGT‐transfected cells; (H) Western blot analysis of OGT and Drp1 expression in cells transfected with si‐OGT and/or piR‐4913 agomir. (I) Western blot analysis of Drp1 levels in aged cardiomyocytes pretreated with MG132 (proteasome inhibitor, 10 µM), chloroquine (CQ, lysosomal inhibitor, 50 µM), or bafilomycin A1 (BafA1, lysosomal V‐ATPase inhibitor, 100 nM) for 6 h, followed by OGT inhibition with OSMI‐1 (20 µM). (J) Drp1 ubiquitination levels in cells treated with OSMI‐1 (20 µM) with or without piR‐4913 agomir. (K) Drp1 O‐GlcNAcylation levels in cells treated with the E1 inhibitor TAK‐243 (20 µM) with or without piR‐4913 agomir. (L, M) Representative immunoblots and quantitative half‐life analysis of endogenous Drp1 in aged cardiomyocytes treated with cycloheximide (CHX, 100 µM) in the presence or absence of piR‐4913 agomir or OGT siRNA. (N, O) Half‐life analysis of exogenous Flag‐Drp1 in aged cardiomyocytes treated with CHX (100 µM) following piR‐4913 agomir and/or OGT siRNA transfection. Data are presented as mean±SEM. ^**^ *p* < 0.01, ^***^ *p* < 0.001.

O‐GlcNAcylation typically regulates protein stability and interactions through the activities of O‐GlcNAc transferase (OGT) or O‐GlcNAcase (OGA). RNA pull‐down assays using biotinylated piR‐4913 confirmed its binding to OGT (Figure 5D). Immunoprecipitation experiments showed that transfection of the piR‐4913 agomir significantly enhanced OGT enrichment on Drp1, whereas transfection of the piR‐4913 antagomir markedly diminished this association (Figure 5E,F). In addition, knockdown of OGT markedly reduced both Drp1 O‐GlcNAcylation and total protein levels; crucially, the piR‐4913 agomir failed to rescue Drp1 expression in OGT‐deficient cells (Figure 5G,H), suggesting that piR‐4913 mediates the O‐GlcNAcylation of Drp1 through OGT and affects its stability.

We further investigated the degradation pathway involved in this process. While the proteasome inhibitor MG132 effectively reversed the OSMI‐1‐induced downregulation of Drp1, inhibitors of the autophagy‐lysosomal pathway (CQ, BafA1) did not (Figure 5I). These results indicate that O‐GlcNAcylation protects Drp1 from the ubiquitin‐proteasome system. Furthermore, pharmacological inhibition of OGT with OSMI‐1 enhanced Drp1 ubiquitination regardless of piR‐4913 status (Figure 5J). Conversely, inhibition of the ubiquitin‐activating enzyme E1 (TAK‐243) did not alter Drp1 O‐GlcNAcylation (Figure 5K), suggesting that O‐GlcNAcylation acts upstream of ubiquitination.

Cycloheximide (CHX) chase assays demonstrated that piR‐4913 overexpression significantly enhanced Drp1 stability, extending its half‐life beyond 24 h. In contrast, OGT knockdown accelerated Drp1 degradation (Figure 5L,M). In cardiomyocytes expressing exogenous Flag‐Drp1, OGT loss markedly shortened the protein half‐life, an effect that was not reversed by piR‐4913 activation (Figure 5N,O). Collectively, these findings demonstrate that piR‐4913 facilitates OGT‐mediated O‐GlcNAcylation of Drp1, which in turn stabilizes the protein by competitively inhibiting its proteasomal degradation.

### 3.6 piR‐4913‐Mediated O‐GlcNAcylation Facilitates Drp1 Mitochondrial Translocation

To determine whether piR‐4913 regulates Drp1 mitochondrial translocation via OGT‐mediated O‐GlcNAcylation, aged cardiomyocytes were transfected with the piR‐4913 agomir under HPostC conditions. Subcellular fractionation and western blot analysis revealed a significant reduction in cytosolic Drp1 with a reciprocal increase in the mitochondrial fraction, while total OGT levels remained unaffected (Figure 6A). Importantly, immunoprecipitation (IP) assays demonstrated that Drp1 localized in the mitochondria exhibited substantially higher O‐GlcNAcylation levels compared to that in the cytoplasm (Figure 6B), suggesting that this modification favors mitochondrial recruitment.

**FIGURE 6:**
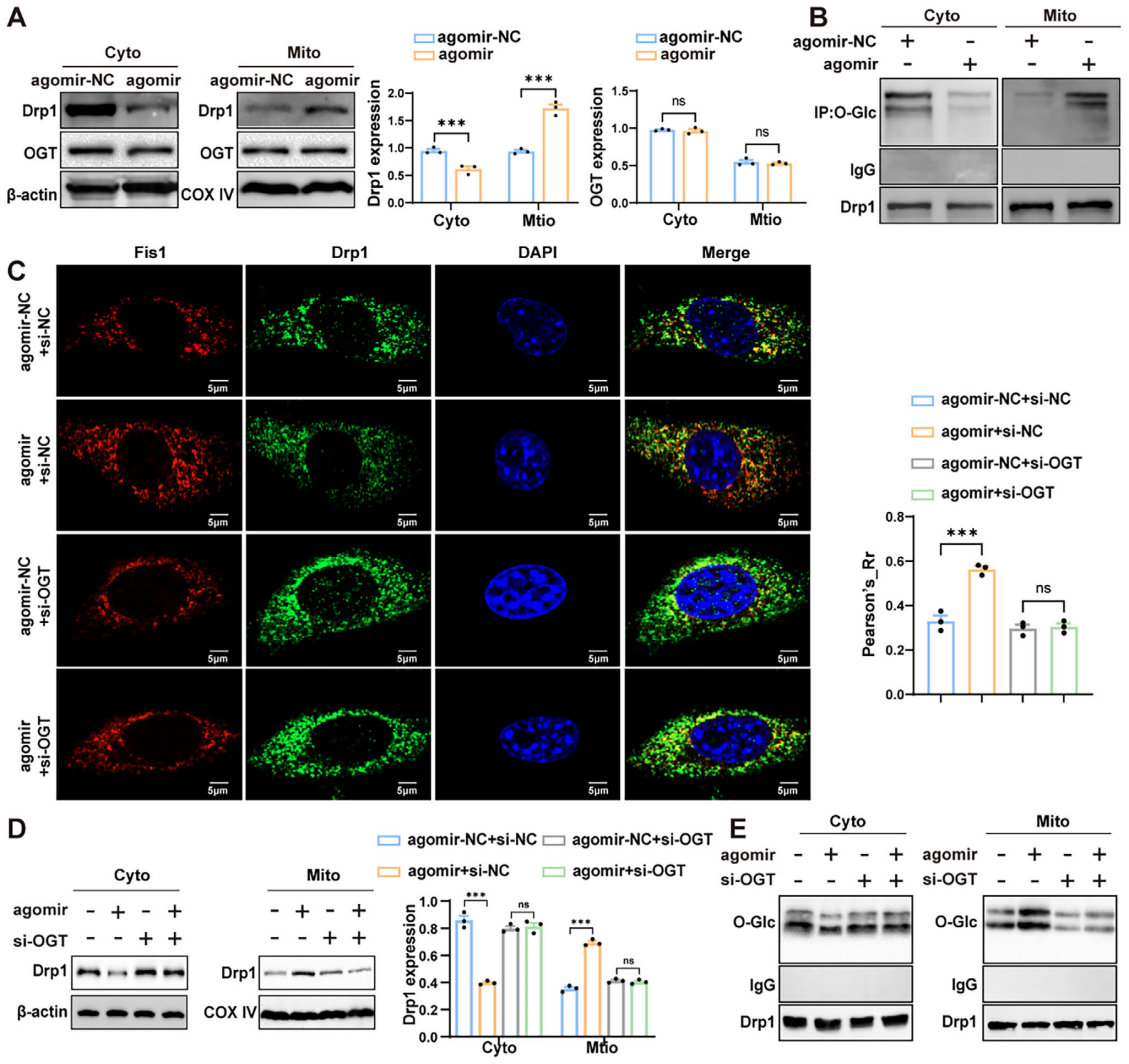
piR‐4913 modulates Drp1 mitochondrial translocation via OGT‐mediated O‐GlcNAcylation. (A) Western blot analysis of Drp1 and OGT protein levels in cytosolic and mitochondrial fractions of aged cardiomyocytes transfected with piR‐4913 agomir under HPostC conditions (*n* = 3). (B) Immunoprecipitation (IP) analysis of Drp1 O‐GlcNAcylation levels in the cytoplasm versus mitochondria. (C–E) Effects of OGT knockdown on piR‐4913‐induced Drp1 redistribution: (C) Representative immunofluorescence images showing the co‐localization of Drp1 (green) with mitochondria (MitoTracker Red, top) and with Fis1 (red, bottom) (*n* = 3). (D) Western blot analysis of Drp1 and OGT expression in cytosolic and mitochondrial fractions following transfection with piR‐4913 agomir and/or si‐OGT (*n* = 3). (E) IP analysis of Drp1 O‐GlcNAcylation levels in the indicated groups. Data are presented as mean±SEM. ^***^ *p* < 0.001.

To validate the necessity of OGT in this process, we performed OGT knockdown in piR‐4913 agomir‐overexpressing cardiomyocytes. Immunofluorescence microscopy showed that OGT depletion significantly attenuated the piR‐4913‐induced recruitment of Drp1 to mitochondria and disrupted the Drp1‐Fis1 interaction (Figure 6C). These findings were corroborated by Western blot and Co‐IP analyses, which confirmed that OGT knockdown abolished the agomir‐induced accumulation of mitochondrial Drp1 and markedly reduced its O‐GlcNAcylation levels in the mitochondrial fraction (Figure 6D,E). Collectively, these data establish that piR‐4913 facilitates Drp1 mitochondrial translocation by enhancing its O‐GlcNAcylation in an OGT‐dependent manner.

### 3.7 O‐GlcNAcylation of Drp1 at T604 Governs its Mitochondrial Translocation and Stability During I/R Injury

To identify the specific O‐GlcNAcylation sites on Drp1, we interrogated the UniProt database and initially identified four candidate residues: T585, T595, T604, and T605 (Figure 7A). Subsequently, we generated a series of Drp1 point mutants (T585A, T595A, T604A, and T605A) to assess their modification status. Immunoblot analysis showed that the T585A and T604A mutations significantly attenuated the O‐GlcNAcylation of Drp1 (Figure 7B). Furthermore, we transfected piR‐4913 agomir and Drp1 point mutants into aged cardiomyocytes. The results showed that only the T604A mutation significantly reversed the promoting effect of piR‐4913 agomir on Drp1 O‐GlcNAcylation. However, the T585A, T595A, and T605A mutations retained a modification level similar to that of wild‐type Drp1 (Figure 7C and Figure S8). Structural studies indicate that Drp1 activity is tightly regulated by nucleotide‐dependent conformational transitions. A structural comparison of the wild‐type Drp1 and the mutant T604A model revealed that the RMSD value for the overall protein alignment was 2.542 Å, indicating a high degree of structural similarity. Subsequently, a detailed analysis of the 540–658 domain revealed that the wild‐type contained three α‐helices, two β‐sheets, and two α‐angles; the T604A mutant contained three α‐helices, two α‐angles, and lacked two β‐sheets. These structural alterations likely impair the protein's functional integrity, potentially compromising its recruitment efficiency to the mitochondria (Figure 7D).

**FIGURE 7:**
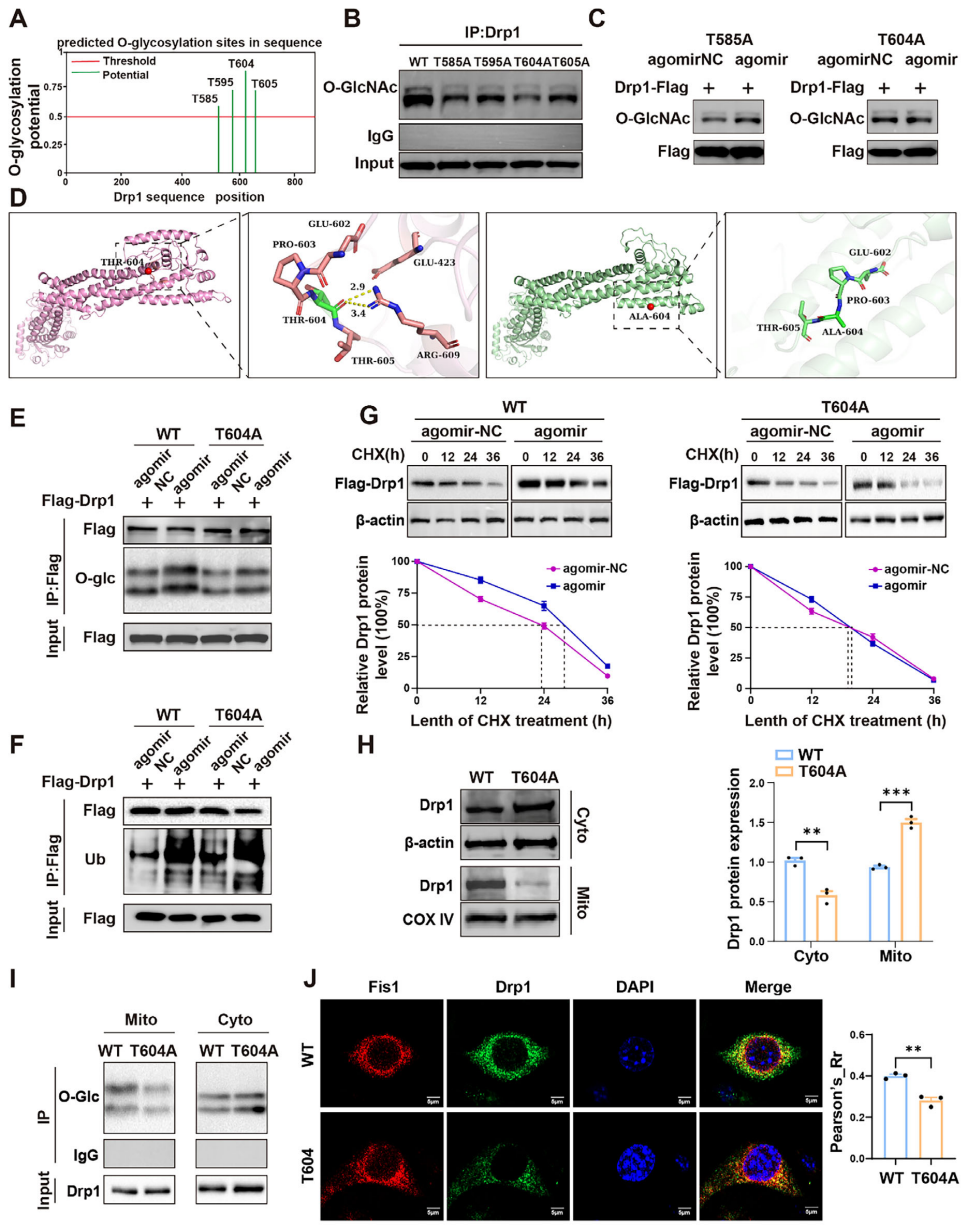
O‐GlcNAcylation at Thr604 governs Drp1 stability and mitochondrial translocation. (A) Bioinformatic prediction of potential O‐GlcNAcylation sites on Drp1 using the O‐GlcNAcylation database. (B) Immunoprecipitation (IP) analysis of O‐GlcNAcylation levels in WT‐Drp1 and various mutants (T585A, T595A, T604A, and T605A) in aged cardiomyocytes under HPostC conditions. (C) IP analysis of O‐GlcNAcylation levels in WT‐Drp1 and various mutants (T585A, T604A) in aged cardiomyocytes transfected with piR‐4913 agomir under HPostC conditions. (D) In silico structural modeling of the Drp1 T604A mutation using AlphaFold3 and conformational comparison with WT‐Drp1 via PyMOL analysis. (E, F) IP analysis of Drp1 ubiquitination and O‐GlcNAcylation levels. (G–J) Effects of the T604A mutation on Drp1 dynamics and stability in aged cardiomyocytes co‐transfected with piR‐4913 agomir: (G) Representative immunoblots and quantitative analysis of Drp1 protein half‐life following cycloheximide treatment. (H) Western blot analysis of Drp1 distribution in cytosolic and mitochondrial fractions (*n* = 3). (I) IP analysis of Drp1 O‐GlcNAcylation levels in mitochondrial fractions. (J) Representative immunofluorescence images showing the co‐localization of Drp1 (green) and Fis1 (red) (*n* = 3). Data are presented as mean±SEM. ^**^ *p* < 0.01, ^***^ *p* < 0.001.

In aged cardiomyocytes, the T604A mutation significantly diminished Drp1 O‐GlcNAcylation while concurrently enhancing its ubiquitination, neither of which was reversed by co‐transfection with the piR‐4913 agomir. (Figure 7E,F). Furthermore, while WT Drp1 exhibited high protein stability (half‐life > 24 h), the T604A mutation markedly accelerated its degradation, regardless of piR‐4913 status (Figure 7G). Subcellular fractionation and immunofluorescence assays confirmed that the T604A mutation impaired Drp1 mitochondrial translocation and weakened its interaction with Fis1 (Figure 7H–J). Collectively, these findings demonstrate that piR‐4913 stabilizes Drp1 and drives its mitochondrial recruitment by modulating O‐GlcNAcylation specifically at the T604 residue.

### 3.8 The Drp1‐T604A Mutation Protects Aged Myocardium Against I/R injury During IPostC

To elucidate the functional significance of Drp1 and its T604 O‐GlcNAcylation site in vivo, we intramyocardially injected AAV9 vectors encoding either wild‐type Drp1 (AAV9‐Drp1^WT^) or the T604 mutant (AAV9‐Drp1^T604A^) into aged rats subjected to I/R and IPostC. Our results demonstrated that the T604A mutation significantly alleviated mitochondrial dysfunction, as evidenced by elevated ATP levels, suppressed ROS production, and preserved mitochondrial membrane potential (MMP) in the aged myocardium (Figure 8A–C). These findings indicate that while Drp1 is a key driver of mitochondrial impairment, the T604A mutation effectively mitigates I/R‐induced organelle dysfunction.

**FIGURE 8:**
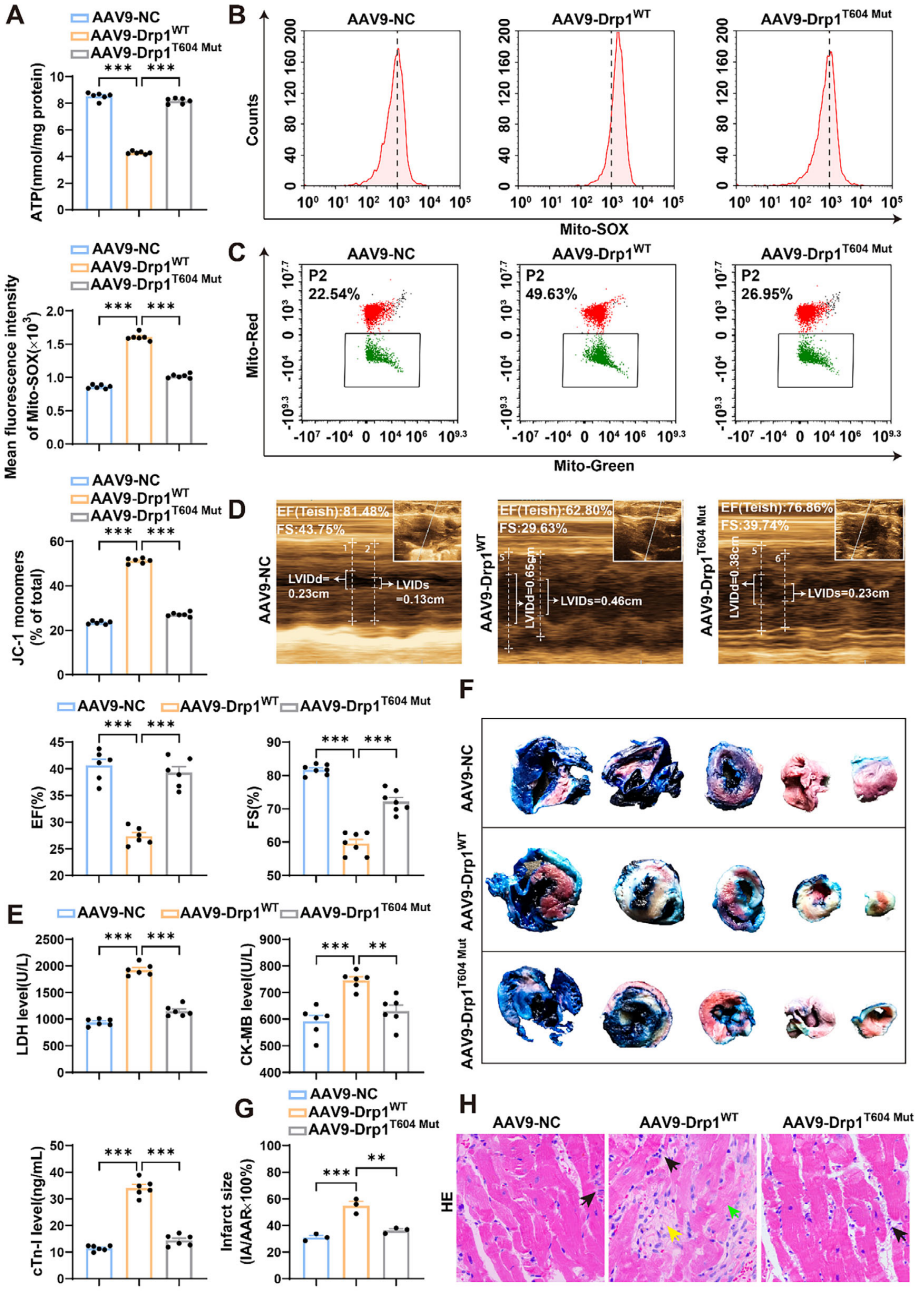
The Drp1‐T604A mutation alleviates I/R injury by restoring mitochondrial function in the aged myocardium. Aged rats were injected intramyocardially with AAV9‐negative control (AAV9‐NC), AAV9‐Drp1^WT^, or AAV9‐Drp1^T604A^ and subsequently subjected to IPostC. (A) Quantitative analysis of mitochondrial ATP production in the aged myocardium (*n* = 6). (B, C) Flow cytometric analysis of mitochondrial ROS levels using the MitoSOX fluorescent probe (B) and mitochondrial membrane potential (MMP) using the JC‐1 probe (C) (*n* = 6). (D) Representative M‐mode echocardiograms (left) and quantitative assessment of left ventricular ejection fraction (EF) and fractional shortening (FS) (right) (*n* = 6). (E) Serum levels of cardiac injury biomarkers (LDH, CK‐MB, and cTn‐I) measured by an automated biochemical analyzer (*n* = 6). (F, G) Representative images of Evans Blue/TTC‐stained heart sections (F) and statistical quantification of myocardial infarct size (G) (*n* = 3). (H) Histopathological changes in the myocardium visualized by HE staining. Scale bar = 50 µm. Data are presented as mean±SEM. ^**^ *p* < 0.01, ^***^ *p* < 0.001.

We subsequently evaluated the therapeutic impact of these constructs on overall myocardial recovery. Echocardiographic assessment revealed that AAV9‐Drp1^WT^ administration exacerbated cardiac dysfunction, characterized by reduced ejection fraction (EF) and fractional shortening (FS); in stark contrast, AAV9‐Drp1^T604A^ significantly improved these contractile parameters (Figure 8D). Consistent with the functional data, the levels of cardiac injury biomarkers (LDH, CK‐MB, and cTn‐I) were markedly elevated in the AAV9‐Drp1^WT^ group but were significantly attenuated by AAV9‐Drp1^T604A^ treatment (Figure 8E). Furthermore, Evans Blue/TTC dual staining confirmed that AAV9‐Drp1^WT^ increased myocardial infarct size, whereas the T604A mutation provided substantial protection, limiting the area of infarction (Figure 8F,G and Figure S9). Histopathological evaluation via HE staining corroborated these findings, showing that AAV9‐Drp1^WT^ worsened myocardial structural disruption and inflammatory infiltration, whereas AAV9‐Drp1^T604A^ preserved tissue integrity (Figure 8H). Collectively, these results demonstrate that the Drp1‐T604A mutation rescues the aged heart from I/R injury by suppressing mitochondrial‐driven pathological processes.

## 4 Discussion

Currently, reperfusion therapy remains the most effective intervention for ischemic heart disease [19]; however, the paradoxical injury it induces necessitates robust adjunctive strategies. As a well‐established cardioprotective strategy, IPostC has been shown to significantly reduce infarct size and preserve cardiac function [2]. In this study, we demonstrate that IPostC restores the mitochondrial fission‐fusion equilibrium by restricting excessive, Drp1‐dependent mitochondrial fragmentation. Crucially, this regulatory process is governed by a novel, cardiac‐enriched piRNA, piR‐4913, which recruits OGT to promote site‐specific O‐GlcNAcylation of Drp1 at Thr604. This post‐translational modification dictates Drp1 mitochondrial translocation and its subsequent docking with Fis1. By reversing this pathological axis, IPostC preserves mitochondrial integrity and cardiomyocyte viability, underscoring the piR‐4913/Drp1 O‐GlcNAcylation cascade as a highly promising therapeutic target for elderly populations.

Mitochondria, the fundamental powerhouses of the cell, dictate cardiomyocyte fate during ischemia and reperfusion [20]. While extensive studies suggest that suppressing excessive fission mitigates I/R injury [8], the precise dynamics in the aged myocardium have remained poorly defined. Aging inherently downregulates key mitochondrial dynamics regulators (e.g., Mfn2, OPA1), creating a baseline vulnerability that exacerbates I/R‐induced mitochondrial fragmentation and dysfunction. Our study reveals that IPostC counteracts this age‐related deficit by effectively blunting pathological Drp1‐mediated fission. By employing specific pharmacological modulators, we confirmed that restraining this excessive fission is an indispensable mechanism for IPostC‐mediated mitochondrial recovery. This finding integrates seamlessly with prior evidence suggesting that IPostC promotes mitochondrial network homeostasis by neutralizing reperfusion‐induced ROS surges and inflammatory cascades. Ultimately, IPostC revitalizes the aged heart not merely by passively reducing injury, but by actively reshaping mitochondrial dynamics to establish a new metabolic equilibrium.

The executors of this complex reprogramming are often non‐coding RNAs [21]. While previous transcriptomic profiling has identified abundant differentially expressed piRNAs in injured or hypertrophic hearts [22], their specific physiological functions, particularly in the senescent myocardium, remain largely enigmatic. Here, we characterized piR‐4913, identifying its crucial, previously unrecognized role in IPostC. Canonically, piRNAs complex with PIWI proteins to silence transposons in the germline. However, our study extends this paradigm: we found that piR‐4913 is predominantly localized in the cytoplasm and binds to target proteins to modulate their functions. During early embryonic development, specific piRNAs regulate the cell cycle and metabolic adaptation under hypoxic stress. Our data suggest that IPostC triggers a response in aged cardiomyocytes that recapitulates certain features of metabolic adaptation, and that piR‐4913 plays a functional role in this process, potentially aiding in the re‐establishment of homeostatic conditions. Unlike other identified piRNAs that exacerbate cardiac injury—such as piR‐HAAPIR [13] (which drives apoptosis) or piR‐000699 [23] (which triggers ferroptosis)—piR‐4913 emerges as a critical node in I/R pathology, and its targeted suppression partly mimics the protective effects of IPostC.

To execute rapid environmental adaptations, these RNA effectors often intersect with PTMs. In the cardiovascular system, O‐GlcNAcylation stands out for its unique metabolic sensitivity [24]. Driven by UDP‐GlcNAc from the hexosamine biosynthetic pathway (HBP), this modification acts as a real‐time nutrient and stress sensor [25]. Through RNA pull‐down coupled with mass spectrometry, we identified the functional interplay between piR‐4913, OGT, and Drp1. While previous reports have established that phosphorylation (e.g., at Ser616 via CaMKII) promotes Drp1 mitochondrial recruitment [26], our findings introduce a critical structural requisite: O‐GlcNAcylation at Thr604. Located within the highly active variable domain of Drp1, this specific modification is dramatically enhanced by piR‐4913. Our mutagenesis studies confirm that the T604A mutation abolishes piR‐4913‐mediated modification, disrupts the active conformation of Drp1, and significantly impairs its oligomerization and binding to Fis1. In the aged myocardium, diminished metabolic flexibility coupled with oxidative stress creates a vicious cycle where pathological O‐GlcNAcylation exacerbates injury. By dynamically regulating Drp1 without altering the genomic sequence, this PTM confers a detrimental “functional plasticity” during acute stress, which IPostC effectively dismantles.

Crucially, because O‐GlcNAcylation is primarily governed by OGT and OGA, it enables hyperacute cellular responses [27]. Our data suggest a fascinating model wherein the 3’‐terminal methylation of piR‐4913 non‐covalently interacts with OGT, structurally enhancing its substrate affinity and catalytic efficiency toward Drp1.

While our study provides a comprehensive roadmap of piRNA‐guided mitochondrial regulation, certain limitations warrant further investigation. First, while we utilized aged rat models and AAV9‐mediated gene delivery to recapitulate the clinical scenario of elderly patients, the inherent biological differences between rodents and humans cannot be overlooked. Human aging is a complex process often accompanied by comorbidities such as hypertension, diabetes, and hyperlipidemia, which may further modulate the piR‐4913/OGT/Drp1 axis in ways not fully captured by our experimental design. Second, our investigation focused primarily on cardiomyocytes; however, myocardial I/R injury is a multicellular pathological process involving endothelial cells, fibroblasts, and infiltrating immune cells. Whether piR‐4913 exerts paracrine effects or regulates mitochondrial dynamics in these non‐myocyte populations remains to be elucidated. Third, although we identified Thr604 as a critical O‐GlcNAcylation site, Drp1 is a highly complex protein subject to a myriad of other post‐translational modifications (PTMs), including phosphorylation, SUMOylation, and S‐nitrosylation. The potential crosstalk between O‐GlcNAcylation and these other PTMs, particularly its interaction with the well‐known Ser616 phosphorylation site under aging conditions, requires further multidimensional proteomic analysis. Finally, although our bidirectional genetic modulation assays demonstrate that piR‐4913 is essential for the association between OGT and Drp1, whether this regulation involves a direct physical three‐body bridge or is assisted by other genes or proteins remains to be fully elucidated by extensive in‑depth studies. Future structural biology studies will be essential to precisely define the non‐covalent interactions between the 3'‐methylated piRNA and the OGT catalytic domain.

In summary, this study reveals a novel mechanism by which IPostC exerts cardioprotective effects in aged hearts. By dismantling the pathological piR‐4913/OGT/Drp1 axis, IPostC restores mitochondrial proteostasis and limits excessive fission. Therapeutically, our data strongly suggest that the application of piR‐4913 antagomirs or targeted small‐molecule inhibitors masking the Drp1 Thr604 site could serve as potent IPostC mimetics. These findings not only broaden our fundamental understanding of piRNA‐guided post‐translational regulation but also provide a precise, mechanism‐driven roadmap for developing next‐generation cardioprotective therapies tailored to the unique metabolic vulnerabilities of the elderly.

## Author Contributions

**Xiangyu Zhao**: methodology, software, data curation, investigation, writing – review and editing, writing – original draft, validation. **Yanchun Li**: methodology, investigation, validation, data curation, writing – review and editing. **Nan Li**: methodology, investigation, data curation, validation, writing – review and editing. **Juan Zhang**: conceptualization, methodology, software, investigation, validation, visualization, writing – original draft, writing – review and editing. **Xiaolong Ma**: conceptualization, methodology, resources, writing – original draft, writing – review and editing. **Fei Yang**: methodology, investigation. **Ruihan Long**: methodology, investigation. **Yining Tian**: methodology, investigation. **Yichen Wang**: methodology, investigation. **Shengchao Ma**: conceptualization, project administration, funding acquisition, writing – review and editing, supervision, validation. **Huiping Zhang**: supervision, conceptualization, funding acquisition, writing – review and editing, project administration, validation. **Yideng Jiang**: conceptualization, supervision, project administration, writing – review and editing, funding acquisition, validation.

## Funding

This work was supported by the grants from the National Major Science and Technology Program (2024ZD0531200); the National Natural Science Foundation of China (82370293, 82270492, 82371598, 82271626, and 82671151); the Key Research and Development Projects in Ningxia Province (2026BEG02029, 2023BEG02074, 2022BFH02013, and 2022BEG02054); the Key Research Projects of Ningxia Medical University (XZ2022005); and the Major Scientific Research Project for High level Health Talents in Hunan Province (R2023120).

## Conflicts of Interest

The authors declare no conflicts of interest.

## Supporting information




**Supporting File 1**: advs78464‐sup‐0001‐SuppMat.docx.

**Supporting File 2**: advs78464‐sup‐0002‐SuppMat.docx.

## Data Availability

The data that support the findings of this study are available from the corresponding author upon reasonable request.
