# Supplementary material for: Ischemic Postconditioning Protects Aged Myocardium Against Ischemia/Reperfusion Injury by Regulating Mitochondrial Dynamics via piR‐4913
Source: Adv Sci (Weinh). 2026 Oct 28:e78464. Online ahead of print. doi: 10.1002/advs.78464 (PMC13715672; doi:10.1002/advs.78464)
Supplement: Supplementary file 1 [file ADVS-9999-e78464-s001.docx]

**Supplementary Figures**

**Supplementary Fig 1. IPostC attenuates I/R injury in the aged myocardium.** **(A)** Schematic diagram illustrating the model of IPostC in aged rats and HPostC in aged cardiomyocytes. **(B)** The changes of electrocardiogram (ECG) were monitored by BL-420N biological signal acquisition and analysis system in aged rats subjected to Sham, I/R and IPostC treatment. Black arrows represent ST segment in lead II; the blue dashed line represents the baseline. **(C)** After mitochondria were purified from the aged myocardium, mitochondrial membrane potential (MMP) was detected by flow cytometry using the JC-1 probe. Red is JC-1 polymer in high potential state, green is JC-1 monomer in low potential state (n=3). **(D)** The levels of LDH, CK-MB, and cTn-I in the culture supernatant of aged cardiomyocytes were detected using ELISA (n=3). **(E)** The viability of aged cardiomyocytes was evaluated by immunofluorescence staining. Green: living cells; red: nuclei of dead cells (n=3). Scale bar=50 µm. **(F)** MMP of aged cardiomyocytes under HPostC was detected by flow cytometry. Red is JC-1 polymer in high potential state, green is JC-1 monomer in low potential state. Data are presented as the mean±SEM. **P*<0.05, ****P*<0.001.

**Supplementary Fig 2. Identification and characterization of aged cardiomyocytes-associated piRNAs**. **(A)** Genomic origin distribution of all detected piRNAs in aged cardiomyocytes (repeat: repetitive regions; gene: gene regions; other: other genomic regions). **(B)** Chromosomal distribution of piRNAs. **(C)** Nucleotide bias at each position of piRNA sequences. **(D)** The gene information and species conservation of piR-4913. (**E**) The distribution of piR-4913 in the nucleus and cytoplasm of aged cardiomyocytes detected by RNA-FISH.

**Supplementary Fig 3. Expression of PIWI proteins in aged cardiomyocytes and myocardium.** The expression of PIWIL1, PIWIL2, PIWIL3, and PIWIL4 in aged cardiomyocytes and myocardium was examined by western blotting.

**Supplementary Fig 4. Transfection efficiency of piR-4913 agomir and antagomir.** After the aged cardiomyocytes were transfected with piR-4913 antagomir followed by Normoxia and H/R, or with piR-4913 agomir followed by HPostC, then **(A-C)** qRT-PCR analysis of piR-4913 expression in aged cardiomyocytes. After the aged rats were injected with piR-4913 antagomir subjected to I/R, or with piR-4913 agomir subjected to IPostC, then **(D-E)** qRT-PCR analysis of piR-4913 expression in aged myocardium. Data are presented as mean±SEM. ***P*<0.01, ****P*<0.001.

**Supplementary Fig 5. The effect of transfection of piR-4913 antagomir on mitochondrial fusion and division in cardiomyocytes under normoxic conditions. (A)** Western blot analysis of mitochondrial fission proteins (Drp1, Fis1) and fusion proteins (OPA1, MFN1, MFN2) in aged cardiomyocytes transfected with piR-4913 antagomir under normoxic conditions (n=3). **(B)** Representative images and quantification of mitochondrial morphology stained with MitoTracker Red (n=3). Scale bars=5 μm. Data are presented as mean±SEM. **P*<0.05.

**Supplementary Fig 6. piR-4913 agomir can disrupt the protective effect of IPostC on myocardial I/R of aged myocardium.** After aged rats were injected with piR-4913 agomir and subjected to IPostC, **(A, B)** Representative M-mode echocardiograms of the LV in short-axis view, along with the measurement of left ventricular EF and FS (n=6). **(C)** Serum levels of lactate dehydrogenase (LDH), creatine kinase-MB (CK-MB), and cardiac troponin I (cTnI) measured by an automated biochemical analyzer (n=6). **(D)** Western blot analysis of mitochondrial fission proteins (Drp1, Fis1) and fusion proteins (OPA1, MFN1, MFN2) in aged myocardium (n=6). **(E, F)** Representative transmission electron microscopy (TEM) images showing mitochondrial ultrastructure in the myocardium (n=6). Scale bar=2 μm. Data are presented as mean±SEM. ****P*<0.001.

**Supplementary Fig 7. Statistics of Evans Blue/TTC staining of the heart from aged rats following administration of piR-4913 antagomir or agomir.** The left ventricular area (LV), area at risk (AAR), and infarct area (IA) were calculated. AAR/LV (%) indicated ischemic region size (n=3).

**Supplementary Fig 8. Comparative analysis of Drp1 O-GlcNAcylation in WT, T595A, and T605A mutants following piR-4913 overexpression.** Immunoprecipitation analysis of O-GlcNAcylation levels in WT-Drp1 and various mutants (T595A, T605A) in aged cardiomyocytes transfected with piR-4913 agomir under HPostC conditions.

**Supplementary Fig 9. Quantification of Evans blue/TTC staining in aged rat hearts from different groups.** The left ventricular area (LV), area at risk (AAR), and infarct area (IA) were calculated. AAR/LV (%) indicated ischemic region size (n=3).
