# Supplementary material for: Ischemic Postconditioning Protects Aged Myocardium Against Ischemia/Reperfusion Injury by Regulating Mitochondrial Dynamics via piR‐4913
Source: Adv Sci (Weinh). 2026 Oct 28:e78464. Online ahead of print. doi: 10.1002/advs.78464 (PMC13715672; doi:10.1002/advs.78464)
Supplement: Supplementary file 2 [file ADVS-9999-e78464-s002.docx]

**Supplementary Materials**

**METHODS**

**piRNA Intervention and Transfection**

The aged cardiomyocytes were transfected with piR-4913 agomir, antagomir, or their respective negative controls (NC) (Genepharma, Shanghai, China) using INVI RNA transfection reagent (Invitrogen, USA) for 24h. The sequences used were as follows:

Agomir: 5′-CAGCUACUCGGGAGGCUGAGACAGGA-3′

Antagomir: 5′-UC CUGUCUCAGCCUCCCGAGUAGCUG-3′,

The transfection efficiency was validated via qRT-PCR.

**Evans Blue/TTC staining**

Myocardial infarct size was determined using Evans Blue and 2,3,5-triphenyltetrazolium chloride (TTC) dual staining. At the end of the 180-min reperfusion period, the left anterior descending artery was re-occluded, and 2 mL of 2% Evans Blue dye (Solarbio, China) was intravenously injected via the femoral vein to delineate the non-ischemic area. The heart was quickly excised, rinsed in saline, and frozen at -20 ℃ for 20 min to facilitate precise sectioning.

The ventricles were sliced into five 2-mm-thick transverse sections using a heart matrix. The slices were then incubated in 1% TTC solution (Solarbio, China) at 37 ℃ for 30 min in the dark to identify viable myocardium. After fixation in 4% Paraformaldehyde Solution (Thermo Scientific, USA) for 24 h, the sections were photographed.

The areas of the left ventricle (LV), the area at risk (AAR, identified as the region not stained by Evans Blue), and the infarct area (IA, identified as the TTC-negative pale region within the AAR) were measured using ImageJ software. The extent of myocardial infarction was expressed as a percentage of the AAR (IA/AAR × 100%).

**Transmission electron microscopy (TEM)**

Myocardial tissues harvested from aged rats were cut into approximately 1 mm^3^ blocks and immediately fixed in 2.5% glutaraldehyde (0.1 M phosphate buffer, pH 7.4) at room temperature for 2 h, followed by overnight incubation at 4 ℃. After three washes with cold phosphate buffer, the samples were post-fixed in 1% osmium tetroxide for 2 h at 4℃. The specimens were then dehydrated through a graded ethanol series (80%, 90%, 95% and 100%), infiltrated with propylene oxide, and embedded in epoxy resin. Ultrathin sections (60-80 nm) were obtained using an ultramicrotome equipped with a diamond knife (Leica Microsystems, Germany), These sections were double stained with 1% uranyl acetate and lead citrate. Finally, the ultrastructure of the myocardium was visualized under a transmission electron microscope (JEM-1400Flash, JEOL, Japan) at an accelerating voltage of 80 kV.

**Hematoxylin and Eosin (H&E) Staining**

Paraffin-embedded myocardial sections (5 µm thick) were deparaffinized in xylene and rehydrated through a graded series of ethanol. The sections were then stained with Mayer’s hematoxylin (Servicebio, China) for 30 s, followed by rinsing in running tap water for 15 min to allow for blueing. Subsequently, the sections were counterstained with eosin (Servicebio, China) for 3 min. After a brief wash, the sections were dehydrated through an ascending ethanol series, cleared in xylene, and mounted with neutral balsam. Pathological changes in the myocardial tissues were observed and captured using a light microscope (Eclipse E100, Nikon, Japan).

**Echocardiography**

Transthoracic echocardiography was performed to evaluate cardiac function using a Vevo High-Resolution Imaging System (FUJIFILM VisualSonics, Toronto, Canada). Rats were anesthetized with 1.5% isoflurane, and their heart rates were maintained within a physiological range during the procedure. M-mode tracings of the left ventricle were obtained from the parasternal long-axis view. Left ventricular internal diameter at end-diastole (LVIDd) and end-systole (LVIDs) were measured. Based on these dimensions, the ejection fraction (EF) and fractional shortening (FS) were automatically calculated using the Vevo software. To ensure objectivity, all measurements were performed by an experienced researcher who was blinded to the experimental groups.

**Serum Biomarker Analysis**

At the conclusion of the 180-min reperfusion period, blood samples were collected from the femoral vein. The samples were allowed to clot at room temperature for 2 h and then centrifuged at 3,000 rpm for 20 min at 4℃ to collect the serum. The serum levels of myocardial injury markers, including lactate dehydrogenase (LDH), creatine kinase-MB (CK-MB), and cardiac troponin I (cTnI), were determined using an automatic biochemical analyzer (Beckman Coulter, Brea, CA, USA) according to the manufacturer’s protocols.

**Enzyme‑Linked Immunosorbent Assay (ELISA)**

The levels of LDH, CK‑MB, and cTn‑I in the cell culture supernatants were quantified using commercially available ELISA kits (Jianglai, Shanghai, China) according to the manufacturer’s instructions. In brief, of each sample or standard was added to the wells and incubated with a biotin-conjugated antibody at 37 ℃ for 60 min. After washing the plates, an enzyme conjugate was added and incubated for 30 min at 37°C. Following another washing step, a substrate solution was added for color development. Finally, the reaction was terminated with 50 μL of stop solution, and the absorbance was measured at 450 nm within 15 min using a microplate reader (Agilent, USA).

**Cell Viability Assay**

Cell viability was evaluated using a Calcein-AM/Propidium Iodide (PI) Double Staining Kit (KeyGen, China). The working staining solution was prepared by adding 1 μL of Calcein-AM and 1 μL of PI to 1 mL of the provided assay buffer, followed by gentle mixing. After being washed with PBS, the cells were incubated with 200 μL of the dye solution for 30 min at 37°C in the dark. Images were captured using a laser scanning confocal microscope (Zeiss, Oberkochen, Germany). Live cells exhibited green fluorescence (Calcein-AM), while dead cells exhibited red fluorescence (PI). The numbers of live and dead cells were counted using ImageJ software, and cell viability was calculated as the percentage of live cells relative to the total cell count.

**ATP Measurement in Myocardial Tissue**

The ATP content in myocardial tissues was determined using a bioluminescence assay kit (Promega, Madison, WI, USA). Briefly, approximately 50 mg of heart tissue was homogenized in 500 μL of ATP assay lysis buffer on ice. The homogenate was then centrifuged at 12,000 g for 5 min at 4℃ to collect the supernatant. Luminescence was measured using a microplate chemiluminescence reader. The absolute ATP concentration was calculated based on a standard curve. To account for variations in sample preparation, the ATP levels were normalized to the total protein concentration of each sample, determined via a BCA protein assay (Beyotime, China), and the results are expressed as nmol/mg protein.

**Oxygen Consumption Rate (OCR) Assay**

Mitochondrial respiration in aged H9c2 cells was evaluated using the Seahorse XF Cell Mito Stress Test Kit on a Seahorse XF24 Extracellular Flux Analyzer (Agilent Technologies, Santa Clara, CA, USA). Cells were seeded in XF24 cell culture microplates at a density of 1.5×10^4^ cells/well. Prior to the assay, cells were equilibrated in XF base medium (supplemented with 1 mM pyruvate, 2 mM glutamine, and 10 mM glucose; pH 7.4) in a CO₂-free incubator at 37°C for 60 min.

The OCR was recorded in real-time under basal conditions and following the sequential injection of mitochondrial inhibitors: oligomycin (1.5 µM), FCCP (1.0 µM), and a mixture of rotenone/antimycin A (0.5 µM). Key parameters, including basal respiration, ATP-linked respiration, and maximal respiratory capacity, were calculated using the Seahorse Wave software. To ensure accuracy, OCR values were normalized to the total protein content of each well determined by a BCA assay.

**Mitochondrial Morphology and Membrane Potential Assessment**

To visualize mitochondrial morphology, cells were incubated with 200 nM MitoTracker Deep Red FM (Thermo Fisher Scientific, Waltham, MA, USA) for 30 min at 37°C. After washing with PBS, mitochondrial structures were visualized using a laser scanning confocal microscope (Zeiss, Germany).

Mitochondrial membrane potential was assessed using a JC-1 Assay Kit (KeyGen, China). Briefly, treated cells or isolated mitochondria were incubated with JC-1 working solution at 37°C for 20 min in the dark. The fluorescence intensities of JC-1 aggregates (red; excitation/emission: 585/590 nm) and monomers (green; excitation/emission: 514/529 nm) were quantified using a flow cytometer (Agilent, USA). The mitochondrial membrane potential was expressed as the ratio of red to green fluorescence intensity.

**High‑Throughput RNA Sequencing**

Total RNA was extracted from aged H9c2 cardiomyocytes using TRIzol reagent (Invitrogen, Carlsbad, CA, USA). The integrity and concentration of the RNA were assessed using an Agilent 2100 Bioanalyzer. Small RNA libraries were constructed through a series of steps: 3' and 5' adapter ligation, cDNA synthesis via reverse transcription, and PCR amplification. The resulting cDNA fragments (representing the small RNA population plus adapters, approximately 150-300 bp) were size selected using gel electrophoresis. After rigorous quality control, the libraries were sequenced on an Illumina HiSeq/MiSeq platform (Personal Biotechnology Co., Ltd., Shanghai, China). Subsequent bioinformatic analysis, including adapter trimming and sequence mapping against piRNA databases (e.g., piRNABank), was performed to identify differentially expressed piRNA profiles.

**Immunofluorescence Staining**

Cells cultured on coverslips were fixed with 4% paraformaldehyde for 30 min at room temperature, followed by permeabilization with 0.5% Triton X-100 for 10 min. To reduce non-specific binding, cells were blocked with 5% normal goat serum (Sigma-Aldrich, USA) for 1 h. Subsequently, the cells were incubated overnight at 4°C with primary antibodies against Drp1 (1:200, Santa Cruz Biotechnology, Dallas, TX, USA) and Fis1 (1:100, Abcam, Cambridge, UK).

After three washes with PBS, cells were incubated with appropriate fluorescein-conjugated secondary antibodies (1:500, ABclonal, Wuhan, China) for 1 h at 37°C in the dark. Nuclei were counterstained with DAPI (Beyotime, China) for 5 min. Fluorescence images were captured using a laser scanning confocal microscope (LSM 800, Zeiss, Oberkochen, Germany). The co-localization of Drp1 and Fis1 was analyzed using ImageJ software.

**Quantitative real-time polymerase chain reaction (qRT-PCR)**

Total RNA was extracted from aged myocardial tissues or H9c2 cells using TRIzol reagent (Invitrogen, USA). The RNA was reverse transcribed into cDNA using the RevertAid First Strand cDNA Synthesis Kit (Thermo Scientific, Waltham, MA, USA). Quantitative PCR was performed using TB Green™ Premix Ex Taq™ (Takara, Tokyo, Japan) on a FTC3000P real‑time PCR system (Funglyn, Toronto, Canada) according to the manufacturer’s instructions.

Specific primers for piR-4913 and other target RNAs were designed and synthesized by RiboBio Co., Ltd. (Guangzhou, China). U6 small nuclear RNA (snRNA) was employed as the internal control for piRNA normalization, while β-actin was used for mRNA normalization. The relative expression levels were calculated using the 2^−ΔΔCt^ method. All experiments were performed in triplicate to ensure reproducibility.

**Western Blot Analysis**

Total Proteins were extracted from aged myocardial tissues or H9c2 cells using RIPA lysis buffer (Beyotime, China) supplemented with a protease and phosphatase inhibitor cocktail. Protein concentrations were quantified using a BCA Protein Assay Kit (Beyotime). Equal amounts of protein (30-50 μg per lane) were separated by 8%-12% SDS-PAGE and then electrotransferred onto PVDF membranes (0.22 or 0.45 μm, Millipore, Billerica, MA, USA).

The membranes were blocked with 5% non-fat milk (BD Biosciences , USA) in TBST for 1 h at room temperature and subsequently incubated overnight at 4°C with primary antibodies against: Drp1 (1:500, Santa Cruz), Fis1 (1:2000, Proteintech), OPA1 (1:10000, Proteintech), Mfn1 (1:1000, Abcam), Mfn2 (1:10000, Proteintech), OGT (1:3000, Abcam), and OGA (1:10000, Abcam).

Following three washes with TBST, the membranes were incubated with HRP-conjugated goat anti-rabbit or anti-mouse IgG (1:4000, ABclonal, China) for 2 h at room temperature. Protein bands were visualized using an ECL substrate (Thermo Fisher, USA) and quantified with Image Lab software (Bio-Rad, Hercules, CA, USA). β-Actin (1:50000, Proteintech) and COX IV (1:20000, Proteintech) served as the loading controls for total and mitochondrial proteins, respectively.

**Co‑immunoprecipitation (Co‑IP)**

Cell lysates were prepared using an IP lysis buffer (Thermo Fisher Scientific, USA) supplemented with a protease inhibitor cocktail and PMSF. After incubation for 30 min at 4 ℃, the lysates were centrifuged at 15,800 g for 15 min. A 100 µL aliquot of the supernatant was reserved as the "Input" control. The remaining supernatant was incubated with 2 µg of anti-Drp1 antibody (Santa Cruz Biotechnology) or a corresponding control IgG (Santa Cruz Biotechnology) overnight at 4 ℃ with constant rotation. Subsequently, the immune complexes were captured by incubation with Protein A/G magnetic beads (Thermo Fisher Scientific, USA) for 2 h at 4 ℃. The beads were then washed three times with cold lysis buffer to remove non-specific binding. The bound proteins were eluted by boiling the beads in SDS loading buffer for 10 min. Finally, the Input, IP, and IgG samples were analyzed by Western blot using specific antibodies against Drp1 and Fis1 to evaluate their physical interaction.

**RNA Immunoprecipitation (RIP)**

RIP assays were performed to investigate the interaction between PIWI protein and piR-4913. Senescent H9c2 cells were lysed in a polysome lysis buffer (containing protease and RNase inhibitors). The lysates were treated with DNase I at 37°C for 10 min, followed by the addition of EDTA, EGTA, and DTT to terminate the reaction. After centrifugation at 12,000 g for 15 min at 4 ℃, a portion of the supernatant was reserved as the Input.

The remaining supernatant was incubated with 5 μg of anti-PIWI antibody or control IgG (ABclonal, China) overnight at 4 ℃ with gentle rotation. Protein A/G magnetic beads (Thermo Fisher, USA) were then added and incubated for an additional 1 h at 4 ℃. The beads were subjected to stringent washes with polysome buffer to minimize non-specific binding. The immunoprecipitated RNAs were extracted using TRIzol reagent and reverse-transcribed into cDNA. The enrichment of piR-4913 was quantified via piRNA-specific qRT-PCR and normalized to the Input levels.

**Fluorescence in situ Hybridization (FISH)**

The subcellular localization of piR-4913 in H9c2 cells was determined using a FISH kit (GenePharma, Shanghai, China). Briefly, cells were fixed with 4% paraformaldehyde for 15 min at room temperature and permeabilized with 0.1% Triton X-100 for 10 min. After blocking, a Cy3-labeled piR-4913 probe (5′‑TCCTGTCTCAGCCTCCCGAGTAGCTG‑3′; Genepharma, Shanghai, China) was denatured and hybridized to the cells in a humidified chamber overnight at 37 °C.

Following hybridization, the cells were washed three times with 2×SSC buffer and once with 1×SSC buffer to remove non-specific binding. Nuclei were counterstained with DAPI (Beyotime, China) for 5 min. Fluorescence images were captured using a laser scanning confocal microscope (LSM 800, Zeiss, Oberkochen, Germany).

**Nuclear‑Cytoplasmic RNA Fractionation**

To determine the subcellular distribution of piR-4913, nuclear and cytoplasmic RNA fractions were isolated from H9c2 cells using a Cytoplasmic & Nuclear RNA Purification Kit (Norgen Biotek, Thorold, ON, Canada) according to the manufacturer’s protocol.

The concentration and quality of the isolated RNA from each fraction were assessed using a spectrophotometer. Subsequently, the RNA was reverse-transcribed into cDNA, and the relative abundance of target RNAs was quantified via qRT-PCR. U6 small nuclear RNA and β-actin mRNA were employed as nuclear and cytoplasmic markers, respectively, to verify the efficiency of the fractionation and for data normalization. The results were expressed as the percentage of the total RNA (nuclear + cytoplasmic) found in each compartment.

**RNA Pull‑down**

To identify the proteins interacting with piR-4913, an RNA pull-down assay was performed using a Biotin RNA Protein Pull-Down Kit (Thermo Fisher Scientific, USA). Briefly, 5′-biotin-labeled piR-4913 probes (5′‑CAGCTACTCGGGAGGCTGAGACAGGA‑3′) and scrambled control probes were synthesized by GenePharma (Shanghai, China). The probes were incubated with streptavidin magnetic beads at 4°C for 30 min to form probe-bead complexes.

H9c2 cell lysates, prepared in a specialized protein-RNA binding buffer supplemented with RNase and protease inhibitors were then incubated with the probe-bead complexes overnight at 4 ℃. After extensive washing to remove non-specific binding, the RNA-bound proteins were eluted by boiling in SDS loading buffer. The enriched proteins were subsequently analyzed by Western blot to verify candidate interactors or by liquid chromatography-tandem mass spectrometry (LC-MS/MS) for de novo protein identification.

**Mass Spectrometry (MS) Analysis**

Proteins captured in the RNA pull-down assays were reduced with 10 mM DTT, alkylated with 50 mM iodoacetamide, and subsequently digested with sequencing-grade trypsin overnight at 37 ℃. The resulting peptides were desalted using C18 ZipTips (Millipore, USA) and analyzed via liquid chromatography-tandem mass spectrometry (LC-MS/MS) on a Q-Exactive HF mass spectrometer coupled to an EASY-nLC 1200 system (Thermo Fisher Scientific, San Jose, CA, USA).

Data were acquired in a data-dependent acquisition (DDA) mode, where each full MS scan was followed by MS/MS scans of the top 20 most abundant precursor ions. The raw data were processed using Proteome Discoverer software (version 2.4, Thermo Fisher Scientific) against the UniProt Rattus norvegicus database. The false discovery rate (FDR) was set to ≤ 1% at both the protein and peptide levels. Proteins identified with at least two unique peptides were considered for subsequent bioinformatic enrichment analysis.

**Material List**

| **Reagents/Consumables/Instruments/Cell Lines** | **Country** | **Company** | **Catalog Number** |
| --- | --- | --- | --- |
| Pentobarbital sodium | USA | Sigma-Aldrich | P3761 |
| H9c2 cells | China | Procell | CL-0089 |
| DMEM | USA | Gibco | 11965092 |
| Fetal bovine serum (FBS) | USA | Invitrogen | 10099141C |
| Penicillin-Streptomycin | USA | Gibco | 15140122 |
| D-galactose | USA | MedChemExpress | HY-B0710 |
| piR-4913 agomir/antagomir | China | Genepharma |  |
| INVI RNA transfection reagent | USA | Invitrogen |  |
| TRIzol Reagent | USA | Invitrogen | 15596026CN |
| RevertAid First Strand cDNA Synthesis Kit | USA | Thermo Scientific | K1622 |
| TB Green™ Premix Ex Taq™ | Japan | Takara | RR420A |
| qRT-PCR Primers for U6/piRNAs | China | RiboBio Co., Ltd. |  |
| Paraformaldehyde Solution | USA | Thermo Scientific | J19943.K2 |
| RIPA Lysis Buffer | China | Beyotime | P0013B |
| BCA Assay Kit | China | Beyotime | P0012 |
| ECL Substrate | USA | Millipore | WBKLS0100 |
| IP Lysis Buffer | China | Beyotime | P0013 |
| Protein A/G Magnetic Beads | USA | Thermo Fisher | 88802 |
| Cytoplasmic RNA Isolation Kit | Canada | Norgen Biotek | 24300 |
| Cytoplasmic & Nuclear RNA Purification Kit | Canada | Norgen Biotek | SKU 21000 |
| Biotin RNA Protein Pull-Down Kit | USA | Thermo Fisher Scientific | 20164 |
| Normal goat serum | USA | Sigma-Aldrich | G9023 |
| Streptavidin Magnetic Beads | USA | Thermo Fisher | 88816 |
| Trypsin (for MS) | USA | Promega | V5280 |
| Non-fat milk | USA | BD Biosciences | 232100 |
| Anti-Drp1 (WB) | USA | Santa Cruz | sc-271583 |
| Anti-Fis1 (WB/IF) | China | Proteintech | 10956-1-AP |
| Anti-OPA1 (WB) | China | Proteintech | 27733-1-AP |
| Anti-Mfn1 (WB) | UK | Abcam | ab221661 |
| Anti-Mfn2 (WB) | China | Proteintech | 13798-1-AP |
| Anti-OGT (WB) | UK | Abcame | ab96718 |
| Anti-OGA (WB) | UK | Abcame | ab124807 |
| HRP-conjugated Goat anti-Rabbit IgG (H+L) | China | ABclonal | AS014 |
| HRP-conjugated Goat anti-Mouse IgG (H+L) | China | ABclonal | AS003 |
| Anti-β-Actin (WB) | China | Proteintech | 66009-1-Ig |
| Anti-COX IV (WB) | China | Proteintech | 11242-1-AP |
| Anti-PIWI (RIP) | China | ABclonal | A3490 |
| Control IgG (RIP/Co-IP) | China | ABclonal | AC005/ AC011 |
| Fluorescein-conjugated Secondary Antibody (IF) | China | ABclonal | AS011 |
| LDH/CK-MB/cTn-I ELISA Kits | China | Jianglai |  |
| Calcein-AM/PI Double Staining Kit | China | KeyGen | KGA331/KGA322 |
| ATP Assay Kit | USA | Promega | FF2000 |
| ZipTips | USA | Millipore | ZTC18M008 |
| Seahorse XF Cell Mito Stress Test Kit | USA | Agilent | 103015-100 |
| Oligomycin, FCCP, Rotenone/Antimycin A (Seahorse) | USA | MedChemExpress | HY-N6782  HY-100410  HY-B1756  HY-N12257 |
| MitoTracker Deep Red FM | USA | Thermo Fisher | M22426 |
| JC-1 Assay Kit | China | KeyGen | KGA601 |
| Evans Blue Dye | China | Solarbio | E8010 |
| 2,3,5-Triphenyltetrazolium Chloride (TTC) | China | Solarbio | T8170 |
| Mayer‘s Hematoxylin | China | Servicebio | G1004 |
| Eosin | China | Servicebio | G1001 |
| Neutral Balsam (Mounting Medium) | China | Servicebio | G1403 |
| IP lysis buffer | USA | Thermo Fisher Scientific | 87788 |
| Vevo High-Resolution Imaging System | Canada | FUJIFILM VisualSonics |  |
| Ultramicrotome equipped with a diamond knife | Germany | Leica Microsystems |  |
| Automatic Biochemical Analyzer | USA | Beckman Coulter |  |
| FTC3000P Real-Time PCR System | Canada | Funglyn |  |
| Microplate Reader | USA | Agilent |  |
| Laser Scanning Confocal Microscope | Germany | Zeiss |  |
| Seahorse XF Analyzer | USA | Agilent |  |
| Flow Cytometer | USA | Agilent |  |
| Illumina HiSeq/MiSeq Platform | USA | Illumina |  |
| Transmission Electron Microscope (JEM-1400Flash) | Japan | JEOL |  |
| Light Microscope (Nikon Eclipse E100) | Japan | Nikon |  |
| Chemiluminescence Reader | USA | Promega |  |
| Q-Exactive HF Mass Spectrometer | USA | Thermo Fisher |  |
| Proteome Discoverer software | USA | Thermo Fisher Scientific |  |
| EASY-nLC 1200 system | USA | Thermo Fisher |  |
| Image Lab software | USA | Bio-Rad |  |
| GraphPad Prism 8.0 software | USA | GraphPad Software |  |
